# Absorption, Stability, and Bioactivity of Fungal-Derived Hyaluronic Acid from *Tremella fuciformis* in a Sequential In Vitro Multi-Barrier Model

**DOI:** 10.3390/foods15071137

**Published:** 2026-03-25

**Authors:** Francesca Uberti, Rebecca Galla, Simone Mulè, Francesca Parini, Claudio Molinari

**Affiliations:** 1 Department for Sustainable Development and Ecological Transition, University of Piemonte Orientale (UPO), 13100 Vercelli, Italy; simone.mule@uniupo.it (S.M.); claudio.molinari@uniupo.it (C.M.); 2 Noivita Srls, Spin Off, University of Piemonte Orientale (UPO), Strada Privata Curti n. 7, 28100 Novara, Italy; rebecca.galla@noivita.it (R.G.); francescaparini00@gmail.com (F.P.)

**Keywords:** fungal-derived hyaluronic acid, *Tremella fuciformis*, oral bioavailability, in vitro digestion and absorption, anti-inflammatory activity, CD44-mediated uptake, hyaluronidase activity

## Abstract

Hyaluronic acid (HA) is widely used in medical, cosmetic, and nutraceutical applications, yet the systemic fate of orally administered HA, particularly non-animal forms, remains poorly characterised. This study investigates the stability, absorption, metabolism, and biological effects of a novel fungal-derived HA extracted from *Tremella fuciformis* using a sequential in vitro multi-barrier model simulating human physiological compartments, including gastric, intestinal, hepatic, renal, chondrocyte, and keratinocyte environments. Across the gastrointestinal stages, fungal-derived HA demonstrated high structural stability, maintained molecular weight, and exerted superior antioxidant and anti-inflammatory activity compared with sodium hyaluronate. It efficiently crossed the intestinal barrier without increasing hyaluronidase activity, indicating protection from premature enzymatic degradation. In hepatic cells, fungal-derived HA exhibited reduced intracellular uptake and greater extracellular persistence, suggesting lower first-pass metabolism and suggesting improved persistence under in vitro conditions. At peripheral targets, it increased the cluster of differentiation 44 (CD44) expression and HA internalisation in chondrocytes and keratinocytes, supporting anti-inflammatory and pro-regenerative effects. Renal assessments revealed minimal excretion and no cytotoxicity, supporting potential systemic availability. Overall, these results provide the first integrated in vitro evidence describing the absorption, distribution, metabolism, and excretion process of fungal-derived HA. This supports the conclusion that this form of HA is stable, biocompatible, and bioactive with therapeutic potential for joint and skin health, as suggested by the in vitro models.

## 1. Introduction

Hyaluronic acid (HA) is a major non-sulphated glycosaminoglycan in the extracellular matrix, characterised by a linear polymer composed of repeating D-glucuronic acid and N-acetyl-D-glucosamine disaccharides joined by alternating β-(1 → 3) and β-(1 → 4) linkages. Besides its structural role in matrix organisation and tissue hydration, HA actively influences cell behaviour, affecting proliferation, migration, and differentiation via receptor-mediated signalling pathways, including CD44 engagement [1]. The natural abundance of this biopolymer in animals and human bodies, along with its biodegradability and biocompatibility, renders HA suitable for the treatment of many diseases [2]. Historically, HA-based therapies have been administered primarily via local or topical routes, such as intra-articular injections for osteoarthritis, dermal fillers for cosmetic medicine, and dressings or hydrogels for wound repair [3]. Evidence suggests that orally administered HA may have wider biological effects, raising questions about its absorption and activity [4].

Elucidating these biological activities requires an assessment of HA’s pharmacokinetic behaviour, including absorption, distribution, metabolism, and excretion. Initially, the maintenance of HA in the digestive tract was questionable. However, studies have shown that HA is acid stable, with minimal degradation in simulated gastric fluids and structural integrity across a range of pH [5,6]. Furthermore, at the intestinal level, HA has been shown to resist enzymatic digestion by epithelial cells and to be partially fermented by the intestinal microbiota, generating oligosaccharides that can improve its absorption [4]. Consistent with these findings, in vitro intestinal barrier models have demonstrated that HA, especially its high-molecular-weight form, can cross epithelial monolayers and support barrier integrity by modulating junctional proteins and inflammatory signalling pathways [7].

The absorption of HA is markedly affected by its molecular weight (MW), with high-MW HA conferring the most significant benefits and exhibiting a rapid turnover rate. Following absorption, HA enters the systemic circulation, where the liver represents the primary metabolic checkpoint. Hepatic clearance involves uptake via sinusoidal endothelial cells and subsequent degradation, mainly mediated by hyaluronidases HYAL1 and HYAL2. Increased hyaluronidase activity has been shown to correlate with liver stress and the generation of low-MW fragments with distinct biological functions [8]. In addition, nly 1% of the HA in the blood is eliminated daily in urine via low-MW fractions that can cross the glomerular barrier in normal conditions [9,10]. Although their contribution is typically considered secondary to that of the liver and other organs, the kidneys may help remove circulating HA, possibly by local uptake or degradation rather than excretion [9]. Thus, characterising HA stability across gastrointestinal, hepatic, and renal environments is essential for understanding its systemic availability.

Finally, HA binds to specific cell receptors and proteins to exert its effects [4]. The primary HA receptor is the multifunctional transmembrane glycoprotein CD44. Almost every type of human cell expresses it in various isoforms, and numerous intracellular signalling pathways that regulate cellular processes are affected by the HA-CD44 interaction [11].

HA, through its physicochemical and signalling properties, contributes to various physiological processes, including tissue repair, maintenance of hydration, regenerative responses, regulation of inflammation, and embryonic development [12]. In this context, HA is important in skin health by binding water, promoting endothelial and fibroblast activity, and supporting collagen formation. As the skin undergoes the process of ageing, cutaneous HA levels decline, resulting in cutaneous dryness, loss of elasticity, and wrinkle formation [13,14]. The efficacy of HA in anti-ageing treatments is attributable to its moisturising, antioxidant and collagen-stimulating properties. The topical and injectable administration of HA is widespread due to its capacity to penetrate the stratum corneum and enhance hydration, smoothness and elasticity. Growing clinical evidence supports the potential of orally delivered HA to enhance skin aesthetic parameters and counteract age-related changes [2,11,15,16,17].

Additionally, several studies over the past few years have shown that HA can be administered orally to counteract skin ageing, thereby enhancing its use in nutraceutical applications [18,19]. Through its role in extracellular matrix structuring, HA helps maintain dermal homeostasis by facilitating proper fibroblast activity [20].

Beyond dermatology, HA maintains musculoskeletal health by being a key part of cartilage and synovial fluid, offering lubrication, shock absorption, and support.

In osteoarthritis, HA degradation results in reduced viscosity and joint dysfunction. Disrupted HA metabolism in arthritic joints leads to reduced MW and concentration, which can worsen inflammation and joint function [21,22]. As demonstrated by clinical research, intra-articular injections of HA have been shown to enhance joint function and alleviate discomfort in individuals afflicted with knee arthritis. Therapeutic efficacy stems from restoring joint HA content, leading to better synovial fluid viscoelasticity and improved lubrication and shock absorption [23]. However, this approach may elicit negative reactions and is not suitable for illnesses involving small joints, such as hand or midfoot arthritis. As an alternative, oral HA treatment has demonstrated promise in both in vivo and clinical studies, reducing knee oedema and inflammation while improving pain and functional outcomes in arthritis [24,25]. In osteoarthritis populations, oral HA intake has shown beneficial effects on pain relief and overall inflammatory status, as indicated by decreases in circulating biomarkers [26].

In addition to animal sources, fungal-derived HA obtained from the fruiting body or extracellular polysaccharides of *Tremella fuciformis* (TFP) has recently attracted attention as a sustainable and easily accessible alternative [27,28].

A range of structural differences in TFPs has been documented, with variation that depends significantly on the source, extraction method, and purification process employed [29]. Reported molecular weights of TFPs vary widely among studies, spanning from 1.08 × 10^3^ to 3.74 × 10^6^ Da [29,30]. Notably, lower-molecular-weight fractions have been associated with enhanced in vitro antioxidant activity [31].

Accumulating evidence highlights the multifunctional roles of TFPs, such as modulating immune responses, antitumour effects, antioxidant and anti-ageing activities, regulating glucose and cholesterol, and neuroprotection [29,30]. Moreover, as demonstrated in the extant literature, TFPs have been shown to reduce free radical levels in vitro [28,31] and to enhance the protective effects of antioxidant enzymes in in vivo models of ageing [32]. This indicates their great potential for managing oxidative stress-induced health problems. HA-like polysaccharides derived from TFP have been shown to exhibit a branched structure, high water-retention capacity, antioxidant properties, and excellent biocompatibility [33]. Indeed, several studies have indicated that these polymers may exhibit superior moisture-binding capacity and protective effects on epithelial and connective tissues compared to bacterial or animal HA [34,35].

In vitro studies with human skin cells, primarily dermal fibroblasts, and biochemical assays have identified various biological activities of polysaccharides extracted from *Tremella fuciformis*. These activities include benefits for skin health [36,37], protection against oxidative stress [37,38], and support for the extracellular matrix [36]. Evidence from simulated gastrointestinal models indicates that TFP maintains substantial digestive stability throughout the oral, gastric, and small-intestinal phases, allowing it to reach the colon as an essentially intact polysaccharide [39]. Once in the colon, human gut microbiota can ferment TFP, altering its physicochemical properties and serving as a substrate for microbial metabolism [40].

This study aimed to investigate the systemic pathway of fungal-derived HA further obtained from a TFP extract using a multistage in vitro model that sequentially reproduces the main physiological processes involved, from digestion to excretion. In this context, the study’s goal was to evaluate the stability and behaviour of HA during passage through the gastric barrier, under simulated digestive conditions, and subsequently through the intestinal barrier, to analyse its absorption. In the first experiment, the metabolic process in the liver was simulated to understand the subsequent fate of HA following absorption. In addition, the biological effects of the compound on target cells, including keratinocytes and chondrocytes, were evaluated. In conclusion, the model facilitated the examination of the mechanisms of HA renal elimination. Concurrently, throughout all phases of the simulated systemic pathway, the stability of HA in terms of titer and molecular weight was meticulously monitored. This approach was adopted to detect any structural alterations or loss of integrity. This integrated approach aims to define, for the first time, the in vitro systemic metabolism and bioaccessibility of a fungal-derived hyaluronic acid. The approach is expected to provide fundamental information to support its potential use in nutraceutical and functional fields.

## 2. Materials and Methods

### 2.1. Agents Preparation

Fungal-derived HA (named GreenIuronic^®^) was obtained from White Tremella (Silver Ear) and was donated by Vivatis Pharma GBHE (Grüner Deich 1–3, 20097 Hamburg, Germany). The manufacturing workflow involves extraction, purification, alcohol-assisted refinement, and physical processing like sieving and particle reduction [30,41,42]. The resulting powder is then packaged, tested for metals, and stored. This powder has previously been characterised to standardise the extract and demonstrate its homology with HA of animal origin [33,35]. Fungal-derived HA was prepared in phenol red–free Dulbecco’s Modified Eagle’s Medium (DMEM; Merck Life Science, Rome, Italy) supplemented with 0.5% foetal bovine serum, 2 mM L-glutamine, and 1% penicillin–streptomycin before experimental assessment. To support mechanistic interpretation, fungal-derived HA (>80% HA; Mw 1500–4000 kDa) was evaluated alongside a fermentation-derived sodium hyaluronate reference (>92% HA; Mw 100–1000 kDa; Merck Life Science, Rome, Italy).

For both agents studied, the concentration used was 0.01%, prepared from a 1% stock solution [34]. The selection of this concentration was informed by prior in vitro studies on Caco-2 cells and a 3D intestinal barrier model, which demonstrated that 0.01% HA provides optimal biological effects while maintaining cell viability and barrier integrity without inducing toxicity [34,35].

### 2.2. Cell Cultures

The human gastric carcinoma GTL-16 cell line (University of Eastern Piedmont, Novara, Italy), Caco-2 colorectal epithelial cells, HepG2 hepatocellular carcinoma cells, T/C-28a2 chondrocytes, HEKa keratinocytes, and HEK293 embryonic kidney cells (ATCC, Manassas, VA, USA) were cultured under standard conditions at 37 °C in a humidified atmosphere containing 5% CO_2_ using Advanced Dulbecco’s Modified Eagle’s Medium–(Adv DMEM) based formulations supplemented with 10% fetal bovine serum, L-glutamine (2 mM), and penicillin–streptomycin (1%).

Prior to treatments, all cell models underwent an 8-h synchronisation step in phenol red–free DMEM without FBS, supplemented with penicillin–streptomycin, L-glutamine, and sodium pyruvate.

GTL-16 and Caco-2 cells were grown on Transwell^®^ inserts (0.4 µm pore size, Corning Costar, NY, USA) to generate gastric and intestinal barrier models. Experimental pH conditions were adjusted to replicate physiological environments (pH 3 for gastric exposure; pH 6.5 apical and pH 7.4 basolateral for intestinal conditions). HepG2 cells were used at passage 2–10 and for liver metabolism analysis at 80–90% confluence, whereas T/C-28a2, HEKa, and HEK293 cells were seeded in multiwell plates as required. Passage ranges were selected based on literature recommendations to maintain phenotypic stability [43,44,45,46,47,48].

### 2.3. Experimental Protocol

**Figure 1:**
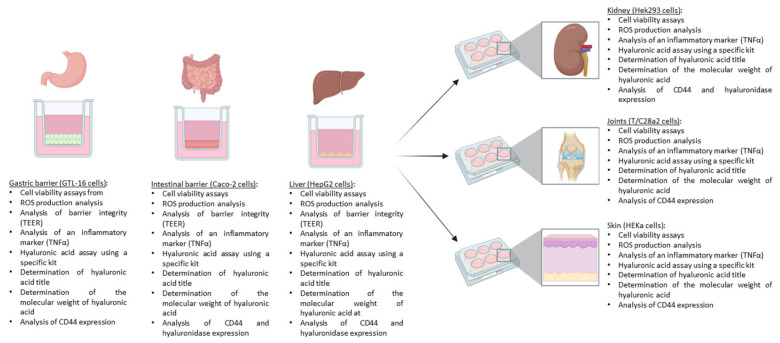
Schematic overview of the sequential in vitro multi-barrier model used to study fungal-derived HA. The workflow simulates sequential physiological compartments: gastric, intestinal, and hepatic phases to assess HA stability, absorption, and metabolism. Conditioned media from the hepatic phase are applied to renal, chondrocyte, and keratinocyte models to evaluate tissue-specific biological responses.

To investigate the metabolic fate of fungal-derived HA and sodium hyaluronate, the experimental strategy was organised into five integrated phases (Figure 1), each corresponding to a specific cellular system. The initial phase involved GTL-16 cells for a time-dependent cytotoxicity assessment over a 1 h – 4 h exposure period. A Transwell^®^ system was used to examine the effects on an in vitro gastric barrier. Experimental outcomes assessed cellular responses, oxidative status, barrier function, inflammation, and HA quantification and characterisation in supernatants.

In the second phase, the basolateral environment of the gastric barrier was successfully used to explore intestinal function from 2 h to 6 h, using Caco2 cells and analysing cell viability, ROS production, barrier integrity, inflammation, and intracellular and extracellular HA, titers, and molecular weight of both substances tested in supernatants. 

In the third step, the basolateral environment of the intestinal barrier was used to explore the hepatic environment in HepG2 cells for 24 h, analysing cell viability, ROS production, barrier integrity, inflammation, and intracellular and extracellular HA titres and molecular weights in the supernatants. 

Finally, in the fourth and last phase, the basolateral environments of the hepatic barrier were used to explore effects on chondrocytes, keratinocytes, and kidney cells for 24 h using T/C-28a2, HEKa, and HEK293 cells, respectively. In each of these compartments, cell viability, ROS production, inflammation, and intracellular and extracellular HA titers and molecular weight were analysed. Also, in the intestinal, hepatic, and renal compartments, hyaluronidase levels were analysed.

All experimental conditions for peripheral targets were normalised to a ‘blank’ sequential control, consisting of culture medium passed through the same barriers without hyaluronic acid, to account for any cytokines or metabolites secreted by upstream cells during the sequential transfer.

### 2.4. In Vitro Gastric Barrier Model

GTL-16 cells were cultured on Transwell^®^ inserts to establish a gastric epithelial barrier model. TEER was monitored for 7 days (EVOM3 voltohmmeter, STX2 electrodes, World Precision Instruments, Sarasota, FL, USA), and experiments were initiated after barrier stabilisation (>150 Ω·cm^2^). The apical compartment was then exposed to acidic conditions (pH 3, 60 min) as previously described [43].

### 2.5. In Vitro Intestinal Barrier Model

An in vitro intestinal barrier was created using Caco-2 cells cultured on Transwell^®^ inserts. Barrier maturation was monitored by TEER measurements over 21 days, and experiments were conducted once resistance values exceeded 400 Ω·cm^2^. Physiological pH conditions were applied prior to stimulation (pH 6.5 on the apical side; pH 7.4 on the basolateral side) to mimic intestinal and systemic environments, followed by TEER verification of barrier stability [35].

### 2.6. In Vitro Liver Barrier Model Using HepG2 Cells

To evaluate hepatic barrier integrity, an in vitro liver model was developed using the HepG2 cell line (ATCC, Manassas, VA, USA). In this setup, cells were plated in the upper chamber of Transwell^®^ culture inserts with a 0.4 µm pore size and cultured in complete growth medium, with regular medium changes to maintain viability and functionality. HepG2 barrier stabilisation was verified by TEER measurement (~486 Ω·cm^2^) using an EVOM3 system prior to experimentation [49]. TEER values were monitored post-exposure to detect any disruptions in barrier integrity.

### 2.7. Cell Viability Assay

Cell viability was assessed using an MTT assay (Merck Life Science, Rome, Italy) following the manufacturer’s instructions [35]. After treatment, cells were incubated with MTT solution, and absorbance was measured at 570 nm (690 nm reference) using a microplate reader. Results were expressed relative to untreated controls across five independent experiments performed in triplicate.

### 2.8. Analysis of ROS Production

Superoxide production was measured using a cytochrome C reduction assay in culture supernatants following standard procedures [50]. Absorbance was recorded at 550 nm, and the results were normalised to untreated controls and expressed as mean ± SD from five independent experiments conducted in triplicate.

### 2.9. Tumour Necrosis Factor α (TNFα) ELISA Kit

TNF-α levels were measured in culture supernatants using a commercial ELISA kit (Merck Life Science, Rome, Italy) following the manufacturer’s instructions [51]. Absorbance was read at 450 nm, and concentrations were determined from a standard curve and expressed relative to untreated controls from five independent experiments performed in triplicate.

### 2.10. CD44 ELISA Kit

CD44 protein expression was measured in cell lysates by ELISA following standard procedures [34]. Briefly, lysates were prepared using radioimmunoprecipitation assay (RIPA) buffer and analysed with a commercial human CD44 ELISA kit (FineTest, Wuhan, China). Absorbance was read at 450 nm, and results were normalised to untreated controls and expressed as mean ± SD from five independent experiments performed in triplicate.

### 2.11. Hyaluronic Acid ELISA Kit

Total hyaluronic acid (HA) levels were measured in cell lysates and culture supernatants using ELISA, following standard procedures [34]. Absorbance was read at 450 nm, and results were normalised to untreated controls, expressed as mean ± SD from five independent experiments carried out in triplicate.

### 2.12. Hyaluronidase (HAase), ELISA Kit

Hyaluronidase (HAase) levels were measured in Caco-2, HepG2, and HEK293 cell lysates using a Human Hyaluronidase ELISA kit (MyBiosource, San Diego, CA, USA) following the manufacturer’s instructions. The absorbance was read at 450 nm with a spectrophotometer (Infinite 200 Pro MPlex, Tecan, Männedorf, Switzerland), and results were normalised to untreated controls and expressed as mean ± SD from five independent experiments performed in triplicate.

### 2.13. Molecular Weight Determination of HA

Hyaluronic acid (HA) molecular weight distribution was determined through 1% agarose gel electrophoresis following established methods [35]. Culture supernatants obtained from GTL-16, Caco-2, HepG2, T/C-28a2, HEKa, and HEK293 cultures were loaded with HA molecular weight standards Select-HA HiLadder and Select-HA Mega Ladder; (Echelon Biosciences, Tebu-Bio Srl, Magenta, Italy) and run under typical electrophoretic conditions. The gels were stained with Stains-All (0.015% in 30% ethanol; Merck Life Science, Rome, Italy), imaged with a ChemiDoc™ Touch system (Bio-Rad, Hercules, CA, USA), and analysed using Image Lab 3.0 software. All samples were run on a single gel; the gel was then carefully sectioned to allow analysis of the results for each compartment.

### 2.14. Uronic Acid-Based Colourimetric Assay for Hyaluronic Acid (HA) Titer

The assay used to quantify HA concentration was the same as that reported in the literature [35]. Uronic acid content in GTL-16, Caco-2, HepG2, T/C-28a2, HEKa, and HEK293 culture supernatants was quantified using a hydroxydiphenyl colourimetric assay following established procedures. Absorbance was measured at 340 nm (Infinite 200 Pro MPlex, Tecan, Männedorf, Switzerland) and concentrations were calculated from a glucuronic acid calibration curve (0–2 mg/mL). Results were expressed as mean ± SD (% *w*/*w*) relative to untreated controls. Unstimulated controls were used to subtract background OD from each sample, ensuring that measured values reflect HA-specific signals only.

### 2.15. Western Blot Analysis

Chondrocyte lysates were prepared in RIPA buffer (50 mM HEPES, 150 mM NaCl, 0.1% SDS, 1% Triton X-100, 1% deoxycholate, 10% glycerol, 5 mM MgCl_2_, and 1 mM EGTA) supplemented with 1 mM NaF, 2 mM sodium orthovanadate, and a protease inhibitor cocktail (1:100; Merck Life Science, Rome, Italy). Equal amounts of protein (40 μg) were separated by Sodium Dodecyl Sulphate-PolyAcrylamide Gel Electrophoresis (SDS–PAGE; 8–15% gels), transferred to polyvinylidene fluoride (PVDF) membranes (GE Healthcare Europe GmbH, Milan, Italy), and probed with primary antibodies against HAS2 and HAS3. Protein expression was normalised to β-actin and expressed as mean ± SD (%).

### 2.16. Statistical Analysis

Data are presented as the mean ± standard deviation (SD) from at least five independent experiments performed in triplicate. Values were normalised to untreated controls. Statistical analyses were conducted using GraphPad Prism 10.2.3 (GraphPad Software, La Jolla, CA, USA), applying one-way ANOVA with Bonferroni post hoc test or Mann–Whitney U test where appropriate. Statistical significance was set at *p* < 0.05.

## 3. Results

### 3.1. Analysis of Fungal-Derived HA in an In Vitro Model of Gastric Barrier

**Figure 2:**
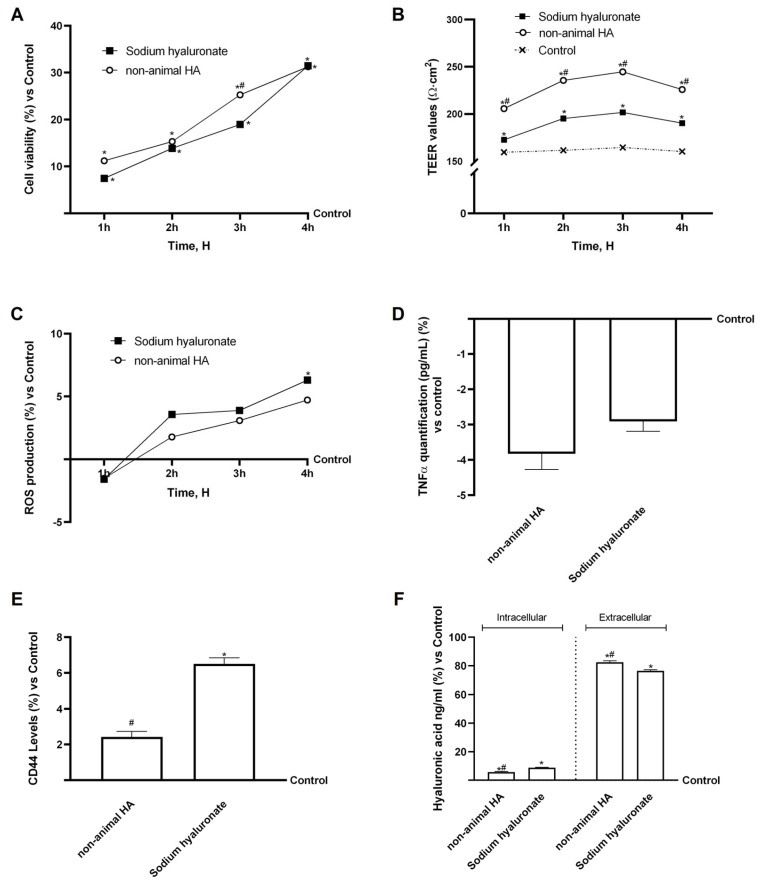
Biological analysis of fungal-derived HA and sodium hyaluronate in the gastric cells (GTL-16). In (**A**) cell viability analysis from 1 to 4 h measured by MTT; in (**B**) integrity analysis through TEER Value using EVOM3; in (**C**) ROS production measured by reduction in cytochrome C from 1 to 4 h; in (**D**) TNFα production analysis at 4 h by specific ELISA kit; in (**E**) CD44 analysis at 4 h by Western blot; in (**F**) HA quantification at intracellular and extracellular levels at 4 h by specific ELISA kit. Data are mean ± SD of five independent experiments performed in triplicate vs. control values (0% line). * *p* < 0.05 vs. control; # *p* < 0.05 vs. sodium hyaluronate.

Initial experiments employed a GTL-16 3D Transwell^®^ (Corning Costar, New York, NY, USA) gastric barrier model to evaluate sample effects under simulated gastric conditions and their passage across the barrier. This preliminary assessment was essential to verify cell viability, epithelial integrity, oxidative stress and inflammatory levels, and to analyse the amount of HA at the intracellular and extracellular levels. As shown in Figure 2A, both fungal-derived HA and sodium hyaluronate maintained cell viability, increasing it compared to the control (*p* < 0.05). Both samples induced a time-dependent increase in cell viability, with fungal-derived HA exerting a greater effect (33% higher than sodium hyaluronate at 3 h, *p* < 0.05). After confirming cell viability, further analyses were performed to evaluate barrier integrity. As shown in Figure 2B, both fungal-derived HA and sodium hyaluronate effectively preserved and enhanced barrier integrity compared to the control (untreated cell, *p* < 0.05). Notably, fungal-derived HA exerted a more pronounced effect than sodium hyaluronate over time, reaching statistical significance (*p* < 0.05). After that, ROS and TNFα production were assessed, as the gastric cell environment is the primary site of contact and metabolism following oral administration of compounds. Alterations in oxidative or inflammatory status at this site may influence both gastric mucosal integrity and the compound’s subsequent systemic bioavailability. As shown in Figure 2C, none of the tested compounds increased ROS production over normal physiological conditions. Specifically, fungal-derived HA maintained a low ROS level throughout all analysed periods, better than sodium hyaluronate, although the difference was not significant. Similar results were obtained in the TNFα production analysis (Figure 2D); especially, both fungal-derived HA and sodium hyaluronate decreased TNFα production compared to the control, but neither significantly. Between the two tested samples, fungal-derived HA induced the greatest decrease in TNFα production. After conducting these safety analyses, the internalisation mechanisms of HA were investigated by analysing the expression of its main receptor, CD44, and quantifying intra- and extracellular HA content (Figure 2E,F). Sodium hyaluronate significantly increased CD44 expression compared with both the control and fungal-derived HA (*p* < 0.05), consistent with the higher proportion of intracellular HA detected following its administration. Conversely, fungal-derived HA induced only a moderate increase in CD44 levels, in line with the higher extracellular HA fraction observed after treatment. Regarding HA determination, a specific ELISA kit was used to verify whether fungal-derived HA was absorbed and internalised as HA or whether it could stimulate endogenous HA production in gastric cells. As shown in Figure 2F, the data showed that the two forms of HA, fungal-derived HA and sodium hyaluronate, were not internalised within the cells and were unable to stimulate endogenous HA production. In this case, fungal-derived HA was less internalised by cells than sodium hyaluronate (*p* < 0.05). Overall, these findings suggest that sodium hyaluronate is taken up more efficiently in the stomach, whereas fungal-derived HA crosses the gastric barrier more efficiently in vitro. In order to confirm that the effect of fungal-derived HA is greater than that of sodium hyaluronate, key biological endpoints (cell viability, ROS production, and CD44 expression) were normalised to the actual amount of pure HA. The results remained consistent (see Table A2 in Appendix A), thereby confirming that the observed differences are not solely attributable to variations in purity.

**Figure 3:**
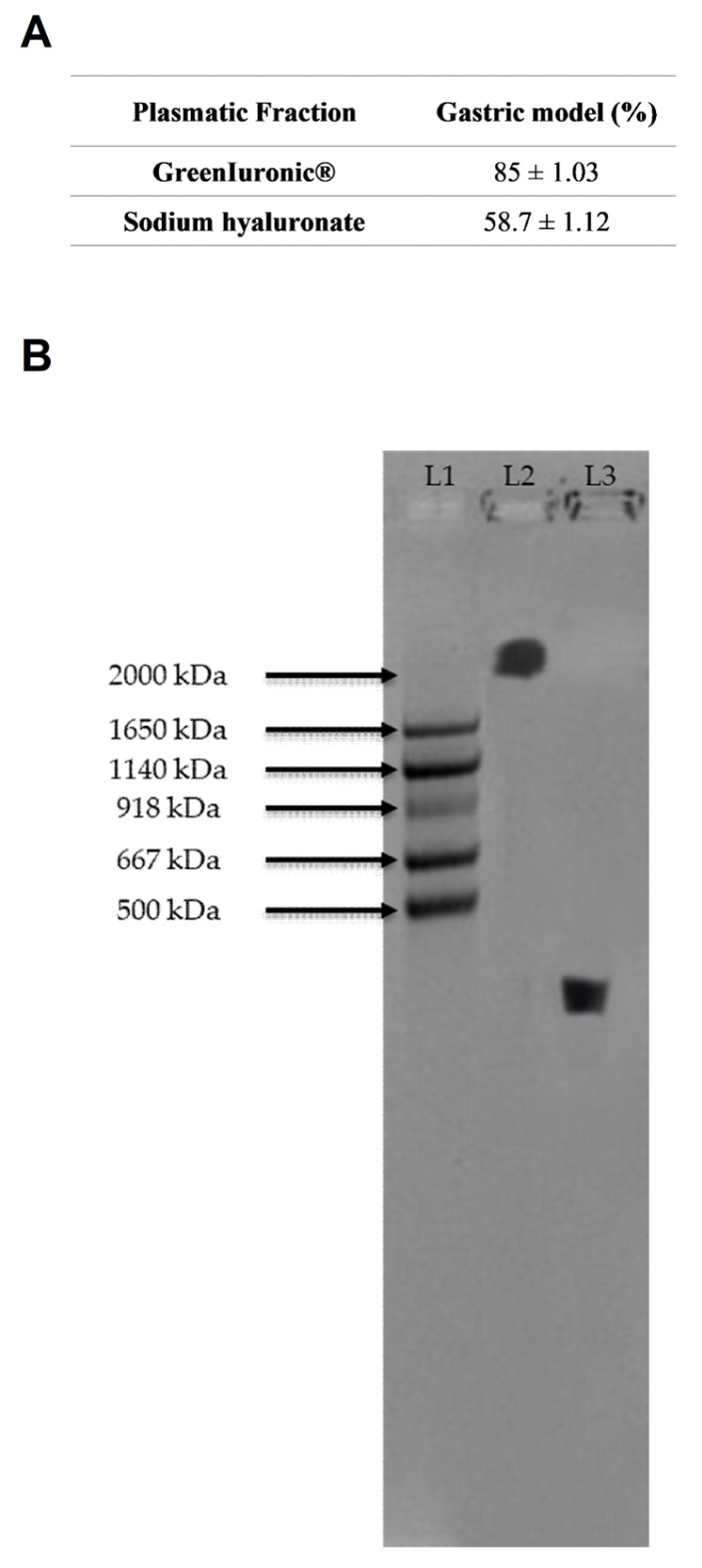
Quantification of HA content and molecular weight in gastric cells (GTL-16). In (**A**), Total HA (% *w*/*w*) was quantified using glucuronic acid calibration curves (0–2 mg/mL) and measured at 340 nm by spectrophotometry (Infinite 200 Pro MPlex, Tecan). Data represent mean ± SD from five independent experiments performed in triplicate. In (**B**), Representative agarose gel (1%) showing HA molecular weight distribution. MW: HA molecular weight standard (Mega + HiLadder); L1: molecular weight ladder; L2: fungal-derived HA; L3: sodium hyaluronate.

In addition, the titre and molecular weight of HA in the gastric supernatant were determined to confirm the previously observed findings. As shown in Figure 3A and in Table A1 (reported in Appendix A), fungal-derived HA had an HA titre of 90.5% before stimulation, while sodium hyaluronate had a titre of 65%. After crossing the gastric in vitro barrier, fungal-derived HA was shown to maintain a higher overall titre than sodium hyaluronate (85% fungal-derived HA vs. 58.7% sodium hyaluronate, *p* < 0.05), indicating greater resistance to enzymatic degradation and the acidic pH of the gastric compartment, with potential bioavailability compared to sodium hyaluronate in the subsequent stages of absorption. Regarding molecular weight (Figure 3B), gel analysis confirmed what was observed previously: fungal-derived HA did not undergo major changes in content or molecular weight, with a molecular weight greater than 2000 kDa.

Overall, these results suggested that fungal-derived HA exhibited good stability in the gastric cell environment. After confirming that fungal-derived HA maintains a high overall concentration after crossing through the gastric barrier, further analysis was performed at the intestinal level.

### 3.2. Analysis of Fungal-Derived HA in an In Vitro Model of Intestinal Barrier

**Figure 4:**
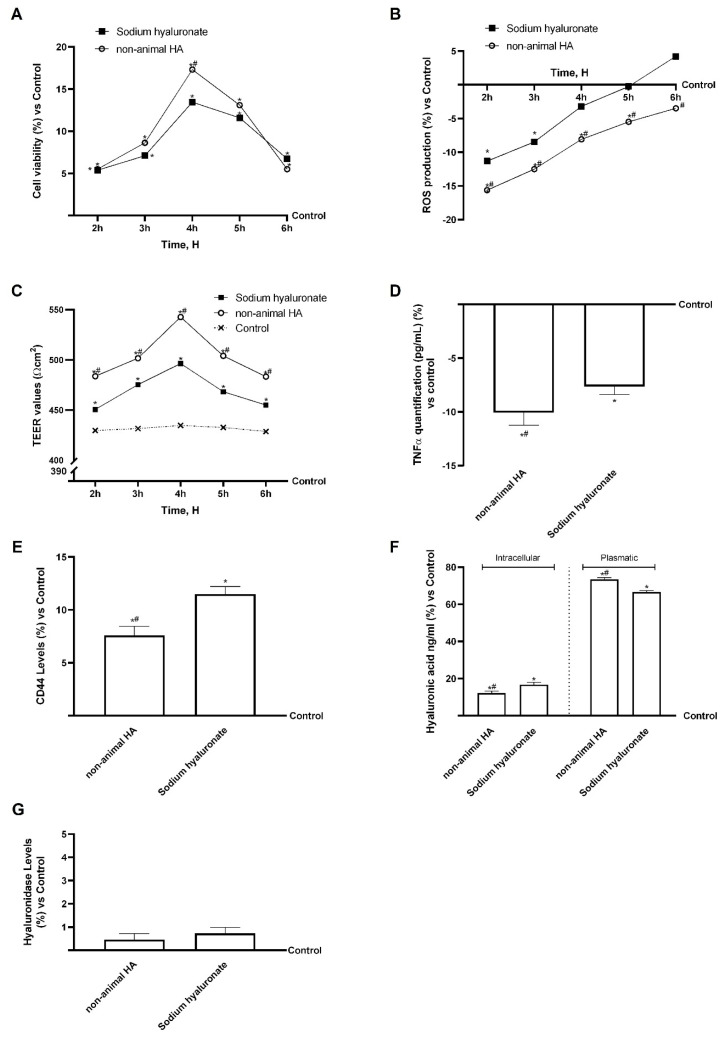
Biological analysis of fungal-derived HA and sodium hyaluronate in the intestinal cells (Caco-2). In (**A**) cell viability analysis from 2 to 6 h measured by MTT; in (**B**) integrity analysis through TEER Value using EVOM3; in (**C**) ROS production measured by reduction in cytochrome C from 2 to 6 h; in (**D**) TNFα production analysis at 6 h by specific ELISA kit; in (**E**) CD44 analysis at 6 h by Western blot; in (**F**), intracellular and extracellular HA quantification at 6 h was determined by ELISA; in (**G**), hyaluronidase levels were measured by ELISA. Data are expressed as mean ± SD from five independent experiments performed in triplicate relative to control (0%). * *p* < 0.05 vs. control; # *p* < 0.05 vs. sodium hyaluronate.

A 3D in vitro intestinal barrier model was used to evaluate safety and intestinal absorption. Gastric eluates containing fungal-derived HA or sodium hyaluronate were applied for 2–6 h, and cell viability, TEER, and ROS production were assessed. In contrast, TNFα production, CD44 levels, and HA concentration were assessed at 6 h. Cell viability peaked at 4 h for both treatments, with fungal-derived HA exhibiting significantly higher values than sodium hyaluronate (~28%, *p* < 0.05). The analysis of TEER (Figure 4B) demonstrates that both the gastric eluate fungal-derived HA and sodium hyaluronate maintained epithelial integrity compared to the control (*p* < 0.05). In particular, gastric eluate fungal-derived HA demonstrates a greater effect than gastric eluate sodium hyaluronate at all times of stimulation (*p* < 0.05). Afterwards, ROS production analysis revealed that gastric eluate fungal-derived HA and sodium hyaluronate were able to induce an even more positive effect (Figure 4C). Gastric eluate sodium hyaluronate was able to maintain ROS levels inferior or equal to the control ones until 5 h after, while gastric eluate fungal-derived HA induced a more positive effect maintaining ROS levels under the control ones even after 6 h of stimulation, demonstrating a greater effect than sodium hyaluronate (*p* < 0.05).

Across the entire observation period, gastric eluate fungal-derived HA showed significantly greater effects than sodium hyaluronate (*p* < 0.05). TNFα production analysis also confirmed the strongest effect of gastric eluate fungal-derived HA over sodium hyaluronate (reduction of about 24%, *p* < 0.05), although both reduced the production compared to the control (Figure 4D, *p* < 0.05). The analysis of CD44 revealed that, also in this case, gastric eluate sodium hyaluronate is more likely to be internalised than gastric eluate fungal-derived HA, as both increase CD44 levels compared to the control (Figure 4E, *p* < 0.05), and sodium hyaluronate increases them further than fungal-derived HA (34%, *p* < 0.05). In addition, the data obtained from the intracellular and basolateral level quantification supported the hypothesis about the higher availability of HA from fungal-derived HA (basolateral environment, Figure 4F). Fungal-derived HA in gastric eluate demonstrated increased translocation through the intestinal barrier, leading to elevated HA levels in the basolateral (plasma-like) compartment compared to control and sodium hyaluronate (~11%, *p* < 0.05).

Furthermore, the study analysed hyaluronidase, an enzyme shown to degrade luminal HA and to be absorbed into the intestinal environment in vitro. The investigation revealed that both fungal-derived HA in the gastric eluate and sodium hyaluronate maintained basal hyaluronidase levels (Figure 4G), with no significant increases compared to the control. This result indicates that neither compound underwent degradation at the cellular level in the intestine. The stable hyaluronidase profile suggests that fungal-derived HA in the gastric eluate can cross the intestinal barrier in vitro without undergoing premature enzymatic hydrolysis.

To account for differences in HA purity between formulations, the main biological endpoints (cell viability, ROS levels, and CD44 expression) were normalised to the effective amount of pure HA, confirming comparable response trends (Table A3, Appendix A).

**Figure 5:**
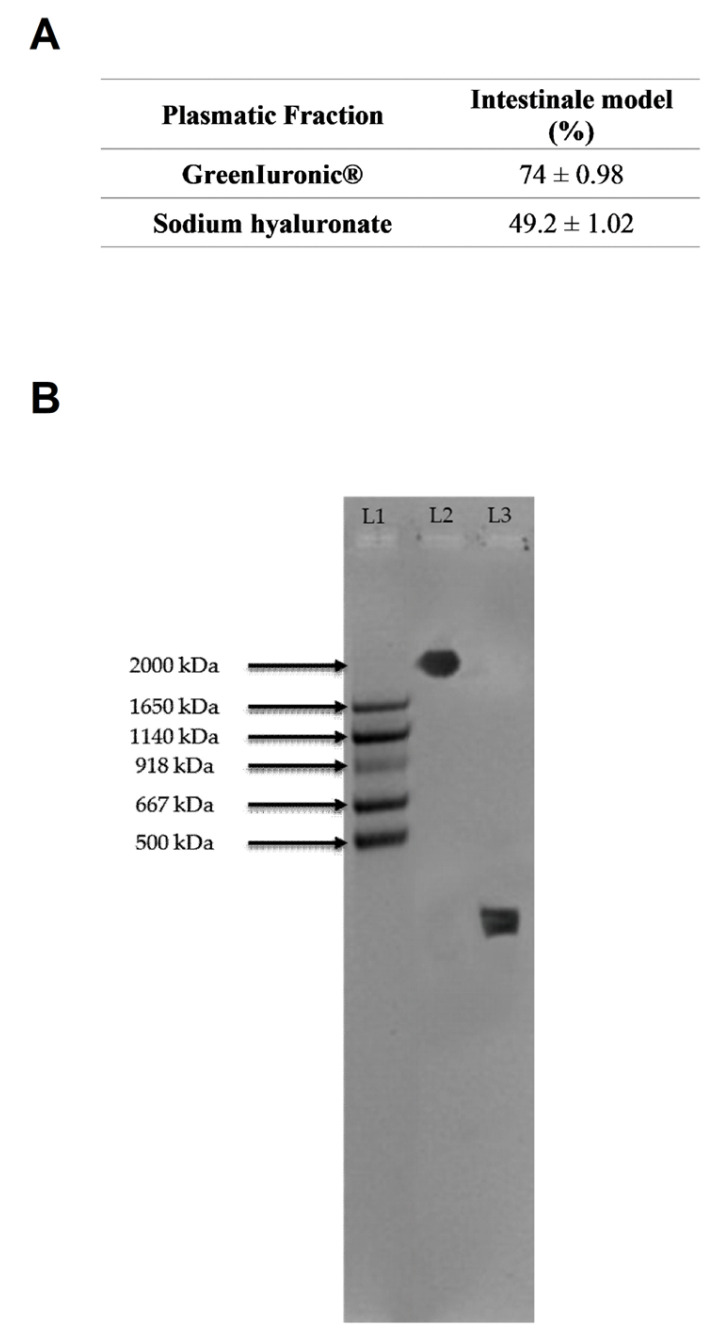
Quantification of HA content and molecular weight in the intestinal cells (Caco-2). In (**A**), Quantification of total HA (% *w*/*w*) based on glucuronic acid calibration curves and spectrophotometric analysis at 340 nm. Data represent mean ± SD from five independent experiments performed in triplicate. In (**B**), Representative 1% agarose gel showing HA molecular weight distribution. MW: HA molecular weight standard; L1: ladder; L2: fungal-derived HA; L3: sodium hyaluronate.

To support the biological data obtained, an analysis of titer and molecular weight in the intestinal supernatant was conducted to ensure the integrity of the tested agents. As shown in Figure 5A and in Table A1 (see Appendix A), gastric eluate fungal-derived HA had an HA titre of 85% after crossing the intestinal in vitro barrier, while gastric eluate sodium hyaluronate had a titre of 58.7%. Fungal-derived HA was shown to maintain a higher overall titre than sodium hyaluronate (74% fungal-derived HA vs. 49.2% sodium hyaluronate, *p* < 0.05). Regarding molecular weight (Figure 5B), gel analysis confirmed what was observed about the titre: fungal-derived HA did not undergo major changes in content or molecular weight, with a molecular weight greater than 2000 kDa.

These findings suggested that fungal-derived HA exhibited greater structural stability and resistance to enzymatic and acidic degradation throughout the gastrointestinal tract, supporting its potential for improved intestinal persistence and subsequent systemic bioavailability compared to conventional sodium hyaluronate. After confirming that fungal-derived HA maintained a high overall concentration after passing through the intestinal barrier, further analysis was performed at the hepatic level using the in vitro intestinal eluate.

### 3.3. Analysis of Fungal-Derived HA in an In Vitro Model of Hepatic Environment

Since the liver represents the primary site of compound metabolism following intestinal absorption, subsequent analyses were performed using hepatic cells.

**Figure 6:**
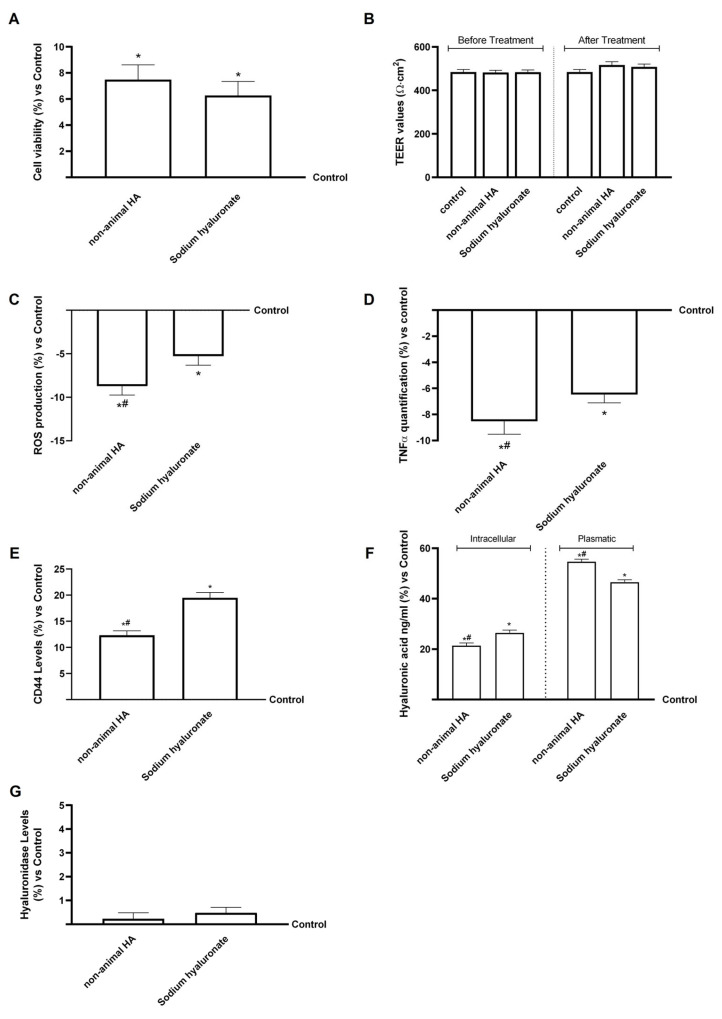
Biological analysis of fungal-derived HA and sodium hyaluronate in the hepatic cells (HepG2). In (**A**), Cell viability (MTT assay); in (**B**), TEER values before and after 24 h treatment; in (**C**), ROS production (cytochrome C reduction assay); in (**D**), TNF-α levels (ELISA); in (**E**), CD44 protein expression (Western blot); in (**F**), Intracellular and extracellular HA levels (ELISA); and in (**G**), Hyaluronidase levels (ELISA). Data are expressed as mean ± SD from five independent experiments performed in triplicate relative to control (0%). * *p* < 0.05 vs. control; # *p* < 0.05 vs. sodium hyaluronate.

As shown in Figure 6A,B, both intestinal eluate fungal-derived HA and sodium hyaluronate significantly increased hepatic cell viability compared with the control (*p* < 0.05), without affecting the integrity of the hepatic layer. In addition, intestinal eluate fungal-derived HA exerts a slightly higher effect on both parameters analysed compared to intestinal eluate sodium hyaluronate (*p* < 0.05). These data confirmed that both compounds were well tolerated by hepatic cells after crossing the gastric-intestinal in vitro barrier.

The evaluation of ROS production (Figure 6C) revealed a significant reduction in oxidative stress in both treatments compared with the control (*p* < 0.05). Notably, intestinal eluate fungal-derived HA induced a more pronounced decrease than intestinal eluate sodium hyaluronate (about 64%, *p* < 0.05), indicating a stronger antioxidant capacity at the hepatic level.

TNFα quantification (Figure 6D) consistently showed that both compounds markedly reduced TNFα production compared with the control (about 31%, *p* < 0.05). Once again, intestinal eluate fungal-derived HA exhibited a significantly greater inhibitory effect than intestinal eluate sodium hyaluronate (*p* < 0.05), highlighting its superior anti-inflammatory potential.

Regarding CD44 receptor expression (Figure 6E), both intestinal eluate fungal-derived HA and sodium hyaluronate upregulated CD44 levels compared to the control (*p* < 0.05), and intestinal eluate sodium hyaluronate showed a significantly higher induction than intestinal eluate fungal-derived HA (*p* < 0.05). This pattern suggested that sodium hyaluronate was taken up more readily by cells, whereas fungal-derived HA remained more available in the extracellular environment. In agreement with these findings, HA quantification in the intracellular and extracellular fractions (Figure 6F) confirmed that intestinal eluate fungal-derived HA led to higher extracellular HA concentrations than the control (*p* < 0.05) and intestinal eluate sodium hyaluronate (about 17%, *p* < 0.05). Conversely, intestinal eluate sodium hyaluronate exhibited higher intracellular HA accumulation, consistent with its increased CD44 expression. In conclusion, given that the liver is a primary site of HA clearance, we analysed hyaluronidase activity in HepG2 cells. Hyaluronidase activity remained stable after both treatments but showed a slight, non-significant reduction upon exposure to fungal-derived HA from intestinal eluate. This trend is consistent with the literature indicating that hepatic hyaluronidases, particularly Hyaluronidase 1 (HYAL1) and Hyaluronidase 2 (HYAL2), mediate early HA degradation and that increased enzymatic activity correlates with tissue stress and early liver damage [52]. The lack of increased hyaluronidase activity in our model suggests that neither form of HA triggered hepatocellular damage or accelerated degradation [53]. Conversely, the reduced activity observed with intestinal eluate fungal-derived HA may partially explain its greater extracellular persistence and slower metabolic turnover in the liver (Figure 6G).

Overall, these results indicate that, at the hepatic level, fungal-derived HA maintains cell viability and exerts antioxidant and anti-inflammatory effects, while promoting a more efficient release of HA into the extracellular compartment, supporting the hypothesis that it can reach several targets outside the hepatocytes. In contrast, sodium hyaluronate tends to accumulate intracellularly, suggesting a different metabolic handling within hepatocytes.

To confirm that fungal-derived HA showed greater activity than sodium hyaluronate, the main biological endpoints (cell viability, ROS production, and CD44 expression) were adjusted according to the actual content of pure HA. The normalised results were consistent (see Table A4 in Appendix A), supporting the conclusion that the differences observed are not merely due to purity discrepancies.

HA concentration and molecular weight distribution were analysed in hepatic supernatants to confirm findings observed in the hepatic model.

**Figure 7:**
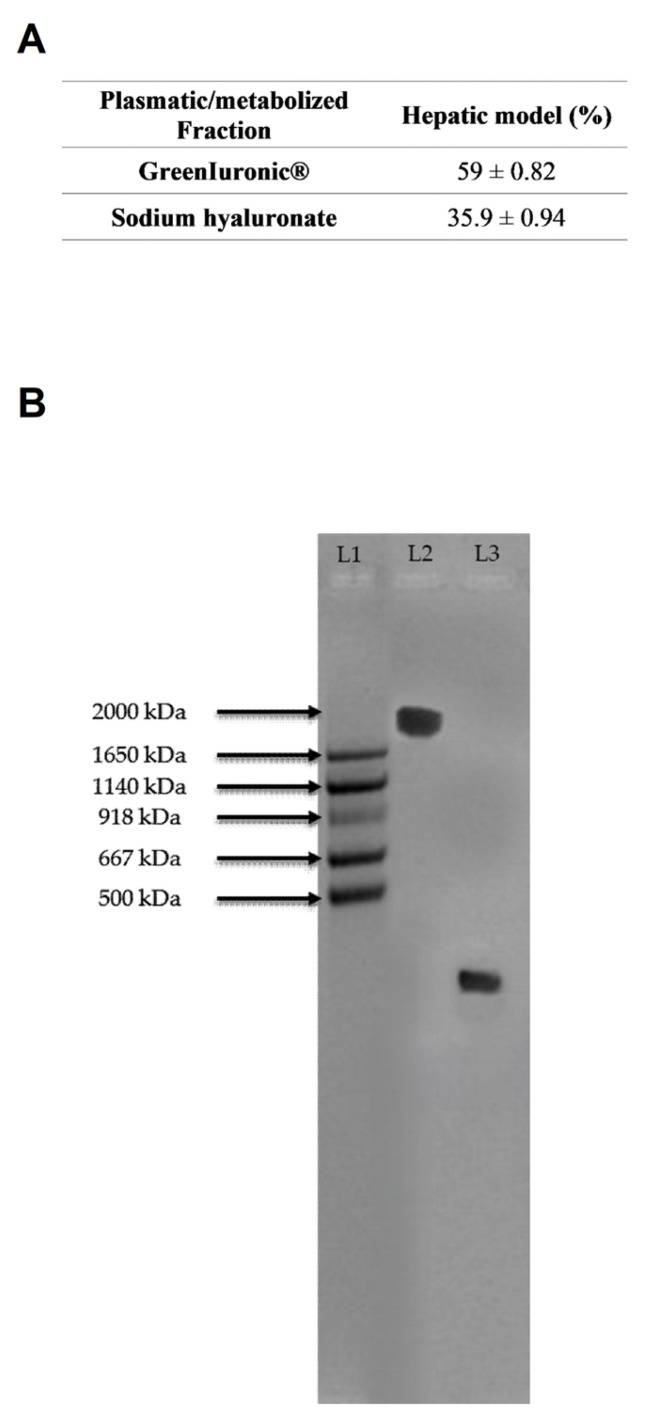
Quantification of HA content and molecular weight in the hepatic cells (HepG2). In (**A**), Total HA quantification (% *w*/*w*) based on glucuronic acid calibration curves measured at 340 nm. Data represent mean ± SD from five independent experiments performed in triplicate. In (**B**), Representative 1% agarose gel showing HA molecular weight distribution. MW: HA molecular weight standard; L1: ladder; L2: fungal-derived HA; L3: sodium hyaluronate.

As shown in Figure 7A and in Table A1 (Appendix A), intestinal eluate fungal-derived HA displayed an HA titre of about 74%, whereas intestinal eluate sodium hyaluronate showed a titre of about 49.2%. Intestinal eluate fungal-derived HA maintained a significantly higher overall titre than sodium hyaluronate (59% vs. 35.9%, *p* < 0.05). However, compared with the initial administration, the titres of both forms of HA were reduced, indicating that a substantial portion of the compounds was metabolised in the hepatocytes and then distributed outside the cells.

Regarding molecular weight (Figure 7B), gel analysis showed that the molecular weights of both HA forms remained consistent throughout the hepatic in vitro barrier (>2000 kDa for fungal-derived HA and <500 kDa for sodium hyaluronate), despite the observed reduction in titre.

Overall, these findings confirm the superior stability and persistence of fungal-derived HA compared with conventional sodium hyaluronate during gastrointestinal and hepatic in vitro transit models. The maintenance of both titre and molecular weight supports the hypothesis that fungal-derived HA may exhibit enhanced systemic bioavailability and prolonged biological activity, likely due to its greater resistance to enzymatic degradation and more controlled release profile. In contrast, sodium hyaluronate appears more susceptible to metabolic degradation, potentially limiting its persistence and systemic efficacy. After confirming that fungal-derived HA maintains a fairly consistent level in the liver, further analyses were performed on kidney cells using the hepatic eluate.

### 3.4. Analysis of Fungal-Derived HA in an In Vitro Model of the Kidney After Gastrointestinal and Hepatic Passage

**Figure 8:**
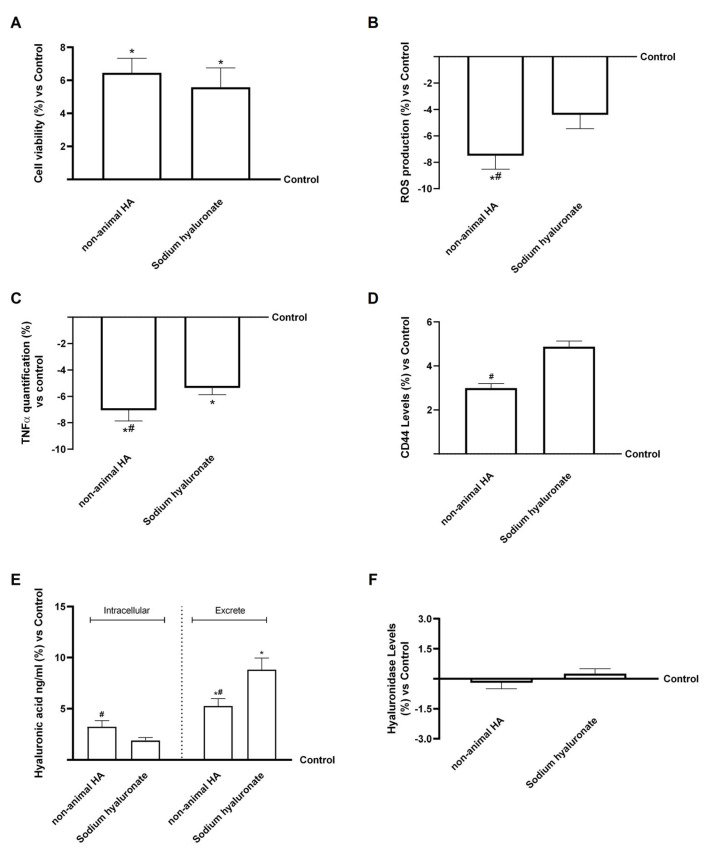
Biological analysis of fungal-derived HA and sodium hyaluronate in the kidney cells (HEK293). In (**A**), Cell viability (MTT assay); in (**B**), ROS production (cytochrome C reduction assay); in (**C**), TNF-α levels (ELISA); in (**D**) CD44 protein expression (Western blot); in (**E**), Intracellular and extracellular HA levels (ELISA); and in (**F**), Hyaluronidase levels (ELISA). Data are expressed as mean ± SD from five independent experiments performed in triplicate relative to control (0%). * *p* < 0.05 vs. control; # *p* < 0.05 vs. sodium hyaluronate.

After hepatic metabolism, the renal processes and excretion of HA were investigated using human renal epithelial, HEK293 cells. This model was used to assess cell viability, oxidative and inflammatory responses, CD44 receptor expression, and intracellular and excreted HA. As shown in Figure 8A, both hepatic eluate fungal-derived HA and hepatic eluate sodium hyaluronate significantly enhanced cell viability compared with the control (*p* < 0.05), confirming their safety in renal cells. The non-animal hepatic eluate HA showed a slightly greater effect, suggesting a favourable interaction with renal epithelial cells.

The evaluation of ROS production (Figure 8B) demonstrated that both compounds significantly reduced oxidative stress relative to control conditions (*p* < 0.05), with hepatic eluate fungal-derived HA exerting a more pronounced antioxidant effect (about 70% more than sodium hyaluronate, *p* < 0.05). These findings suggested that fungal-derived hepatic eluate HA contributed to maintaining redox homeostasis in renal cells, a key factor in preventing damage. Again, analysis of TNFα production (Figure 8C) revealed a significant decrease in its production following treatment with both samples compared with the control (*p* < 0.05). Once again, the fungal-derived hepatic eluate HA showed a stronger anti-inflammatory effect (approximately 30% greater than sodium hyaluronate, *p* < 0.05), consistent with its protective activity observed in other cellular models.

Regarding CD44 (Figure 8D), both the fungal-derived hepatic eluate HA and sodium hyaluronate showed a stronger anti-inflammatory effect (approximately 30% greater increase than the control, even if not significant), confirming receptor engagement and HA internalisation. Sodium hyaluronate showed slightly higher CD44 upregulation, consistent with greater cellular uptake. Importantly, the HA quantification analysis (Figure 8E) provided key insights into renal cellular function. Both compounds increased intracellular HA compared with the control, even if not significantly; however, hepatic eluate sodium hyaluronate exhibited a markedly higher proportion of HA in the extracellular compartment (*p* < 0.05). Finally, hyaluronidase activity was also quantified (Figure 8F). Hepatic eluate fungal-derived HA and sodium hyaluronate all maintained basal hyaluronidase levels, with fungal-derived HA showing a modest decrease compared to the control. In the kidney, hyaluronidase-mediated degradation of interstitial HA is closely related to water reabsorption dynamics, particularly under antidiuretic hormone (ADH) stimulation [54]. The lack of enzymatic activation in our model suggests that neither form of HA altered renal HA turnover or water regulatory pathways. Taken together, these results demonstrate that fungal-derived HA maintains renal cell viability and reduces oxidative and inflammatory stress. The lower extracellular HA levels observed after fungal-derived HA treatment suggest that it is more efficiently absorbed during the passage through the gastric-intestinal-hepatic in vitro barriers.

In order to provide further substantiation for the greater activity of fungal-derived HA in comparison with sodium hyaluronate, the key biological endpoints (cell viability, ROS production, and CD44 expression) were normalised to the effective amount of pure HA. The adjusted results remained consistent (see Table A5 in Appendix A), indicating that the observed differences are unlikely to be attributable solely to variations in purity.

**Figure 9:**
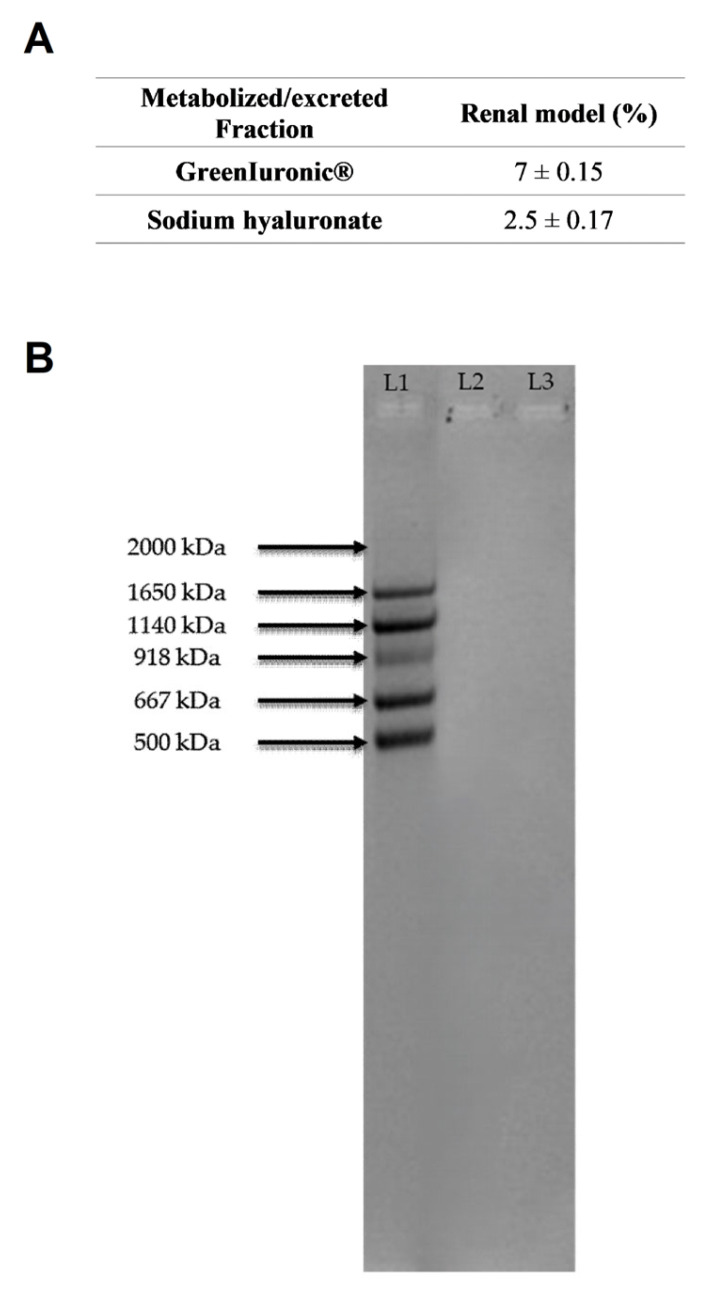
Quantification of HA content and molecular weight in the kidney cells (HEK293). In (**A**), quantification of total HA (% *w*/*w*) is based on glucuronic acid calibration curves measured at 340 nm. Data represent mean ± SD from five independent experiments performed in triplicate. In (**B**), a representative 1% agarose gel shows HA molecular weight distribution. MW: HA molecular weight standard; L1: ladder; L2: fungal-derived HA; L3: sodium hyaluronate.

Also in the excretion compartment, to confirm what was observed biologically in renal cells, the titre and molecular weight of HA in the HEK293 cells supernatant were determined. As shown in Figure 9A and in Table A1 in Appendix A, hepatic eluate fungal-derived HA displayed an HA titre of 59% after intestinal passage. In contrast, hepatic eluate sodium hyaluronate showed a titre of 35.9%. After renal passage, fungal-derived HA showed a higher overall titre compared with sodium hyaluronate (7% vs. 2.5%, *p* < 0.05), although both values were drastically reduced relative to the hepatic compartment. Nevertheless, the residual titre observed for fungal-derived HA suggested that a measurable fraction of the compound remained intact and was excreted as high-molecular-weight HA, confirming its greater stability during all intracellular passages. The higher renal recovery of fungal-derived HA compared with sodium hyaluronate further supports the hypothesis that this formulation possesses improved resistance to enzymatic degradation.

Regarding molecular weight at the renal level (Figure 9B), it was not possible to determine the molecular weight of either hepatic eluate HA form due to the very low HA content in both samples. Nevertheless, fungal-derived HA exhibited a higher overall titre than sodium hyaluronate (7% vs. 2.5%, *p* < 0.05), indicating greater persistence.

Overall, these results suggest that fungal-derived HA is more resistant to metabolic degradation than sodium hyaluronate, maintaining a measurable fraction in the renal compartment, which supports its potential for prolonged bioavailability and delivery to other target tissues, as tested using hepatic eluate.

### 3.5. Effects of Fungal-Derived HA on Chondrocytes After Gastrointestinal and Hepatic Passage

Since exogenous HA has a direct biological effect on chondrocytes when administered into articular cartilage, several experiments were carried out to explore the effects of fungal-derived HA from hepatic eluate, compared to sodium hyaluronate from hepatic eluate, on chondrocyte viability, ROS production, TNFα production, and HA internalisation.

**Figure 10:**
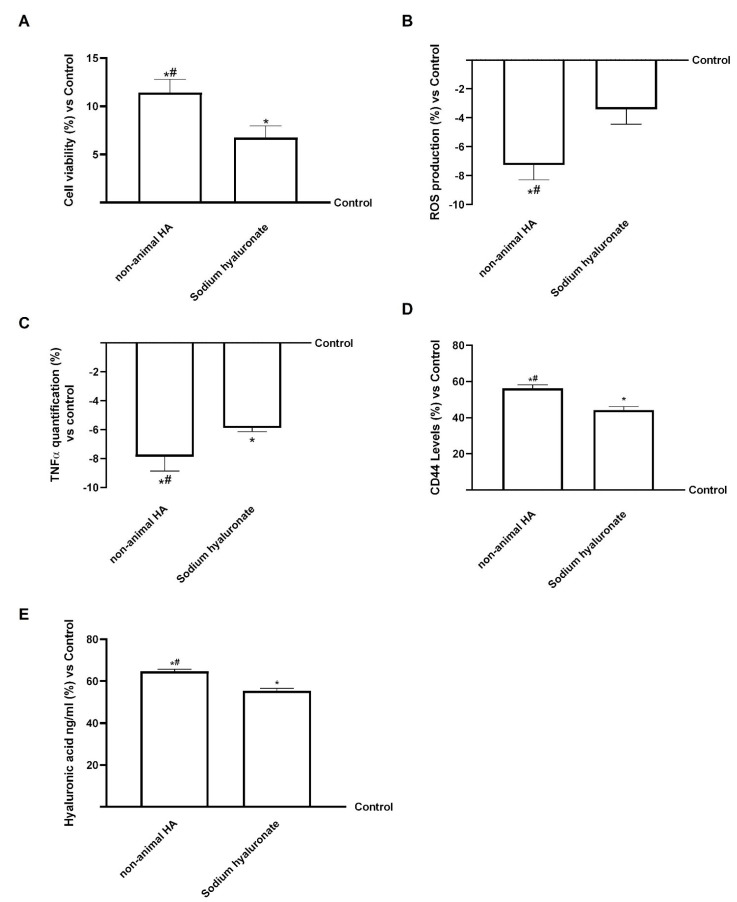
Biological analysis of fungal-derived HA and sodium hyaluronate in chondrocyte cells (T/C28a2). In (**A**), cell viability (MTT assay); in (**B**), ROS production (cytochrome C reduction assay); in (**C**), TNF-α levels (ELISA); in (**D**), CD44 protein expression (Western blot); in (**E**), intracellular and extracellular HA levels (ELISA). Data are expressed as mean ± SD from five independent experiments performed in triplicate, relative to control (0%). * *p* < 0.05 vs. control; # *p* < 0.05 vs. sodium hyaluronate.

Both hepatic eluate fungal-derived HA and sodium hyaluronate markedly enhanced chondrocyte viability compared with the control (*p* < 0.05; Figure 10A). Fungal-derived HA exhibited a slightly higher effect than sodium hyaluronate, confirming its good cellular biocompatibility and potential trophic activity on cartilage cells. Further, the analysis of ROS production (Figure 10B) revealed that both compounds exerted antioxidant effects, reducing oxidative stress relative to the control (*p* < 0.05). Again, from hepatic eluate, fungal-derived HA induced a more pronounced decrease than sodium hyaluronate (about 1.1-fold, *p* < 0.05), indicating a stronger capacity to maintain redox balance within chondrocytes. Similarly, TNFα quantification (Figure 10C) demonstrated that both hepatic eluate fungal-derived HA and sodium hyaluronate significantly reduced TNFα release compared with the control (*p* < 0.05), with fungal-derived HA showing a superior anti-inflammatory effect compared to sodium hyaluronate (about 34%, *p* < 0.05). This result suggests that fungal-derived HA may help to limit inflammatory processes typically involved in cartilage degradation in vitro.

CD44 analysis (Figure 10D) showed significant upregulation after treatment with both samples compared to control (*p* < 0.05), with hepatic eluate fungal-derived HA causing a greater increase than sodium hyaluronate (~27%, *p* < 0.05). This finding is particularly relevant, as CD44 is the main receptor mediating HA internalisation and downstream signalling in chondrocytes, and an increase in CD44 indicates that HA is properly internalised into chondrocytes. In agreement with these results, HA quantification (Figure 10E) revealed a significantly greater intracellular HA concentration following treatment with fungal-derived HA hepatic eluate compared with both sodium hyaluronate and the control (about 17% vs. sodium hyaluronate, *p* < 0.05). The enhanced internalisation of fungal-derived HA suggests improved interaction with chondrocyte HA receptors and potentially stronger stimulation of intracellular pathways involved in matrix synthesis and tissue repair in vitro. Indeed, this enhanced internalisation is beneficial, as it supports HA bioactivity within the cells, possibly contributing to the restoration of cartilage homeostasis and the maintenance of extracellular matrix integrity.

To further support the higher activity of fungal-derived HA compared with sodium hyaluronate, the main biological endpoints (cell viability, ROS production, and CD44 expression) were normalised to the actual amount of pure HA. The corrected data remained consistent (see Table A6 in Appendix A), suggesting that the observed differences are unlikely to be explained solely by differences in purity.

**Figure 11:**
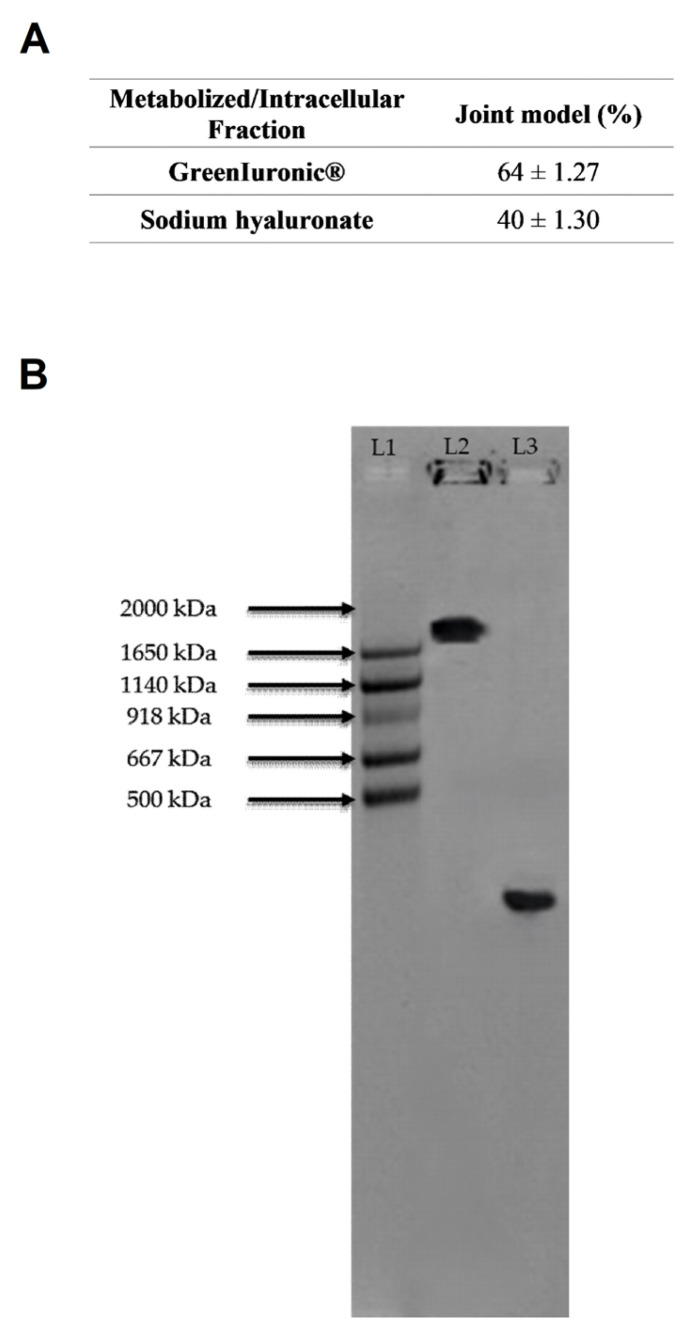
Quantification of HA content and molecular weight in the chondrocyte cells (T/C28a2). In (**A**), total HA quantification (% *w*/*w*) based on glucuronic acid calibration curves measured at 340 nm. Data represent mean ± SD from five independent experiments performed in triplicate. In (**B**), representative 1% agarose gel showing HA molecular weight distribution. MW: HA molecular weight standard; L1: ladder; L2: fungal-derived HA; L3: sodium hyaluronate.

Following biological analyses at the chondrocyte level, the titre and molecular weight of the fungal-derived HA and sodium hyaluronate in the intracellular fraction of chondrocytes were determined. From the data shown in Figure 11A and Table A1 (Appendix A), it was determined that the titre of the fungal-derived HA in the hepatic eluate was approximately 64%. In contrast, that of hepatic eluate sodium hyaluronate was approximately 40%. This difference may arise from the fact that fungal-derived HA seems to induce greater endogenous production than sodium hyaluronate. To confirm that the molecular weight analysis was performed. As shown in Figure 11B, hepatic eluate fungal-derived HA maintains its high molecular weight, whereas hepatic eluate sodium hyaluronate shows only a minimal change in molecular weight.

Overall, these results indicate that fungal-derived HA not only maintains its structural integrity and high molecular weight during in vitro metabolism but also appears to stimulate endogenous HA synthesis at the chondrocyte level. This dual effect, preservation of exogenous HA and induction of endogenous production, suggests a more sustained and efficient contribution to joint matrix homeostasis compared to conventional sodium hyaluronate.

**Figure A1:**
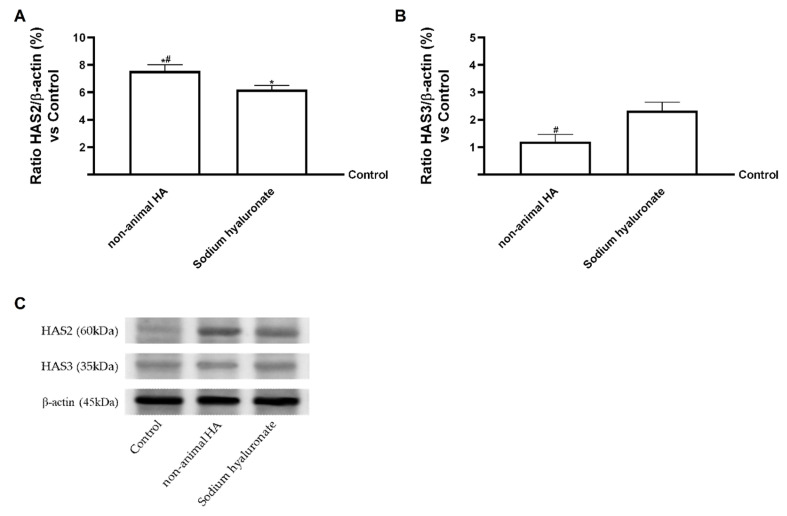
Expression of hyaluronan synthases (HAS) isoforms in chondrocytes after treatment with both hyaluronic acid form. In (**A**), HAS2 expression evaluated with Western Blot analysis; in (**B**), HAS3 expression quantified with Western Blot analysis; and in (**C**), an example of Western Blot lane. * *p* < 0.05 Vs. Control; # *p* < 0.05 Vs. Sodium hyaluronate.

In order to confirm the effective endogenous production of HA in chondrocytes, analysis of HAS revealed a significant upregulation of HAS2 expression in chondrocytes treated with fungal-derived HA compared to control (*p* < 0.05), while HAS3 levels remained unchanged (Figure A1 in Appendix A). The results obtained demonstrate that the increased HA content observed in the joint model is associated with the selective activation of the HAS isoform, with a greater increase in HAS2 expression of approximately 18% and a reduction in HAS3 expression by about 49% compared to sodium hyaluronate (*p* < 0.05).

### 3.6. Effects of Fungal-Derived HA on Keratinocytes After Gastrointestinal and Hepatic Passage

**Figure 12:**
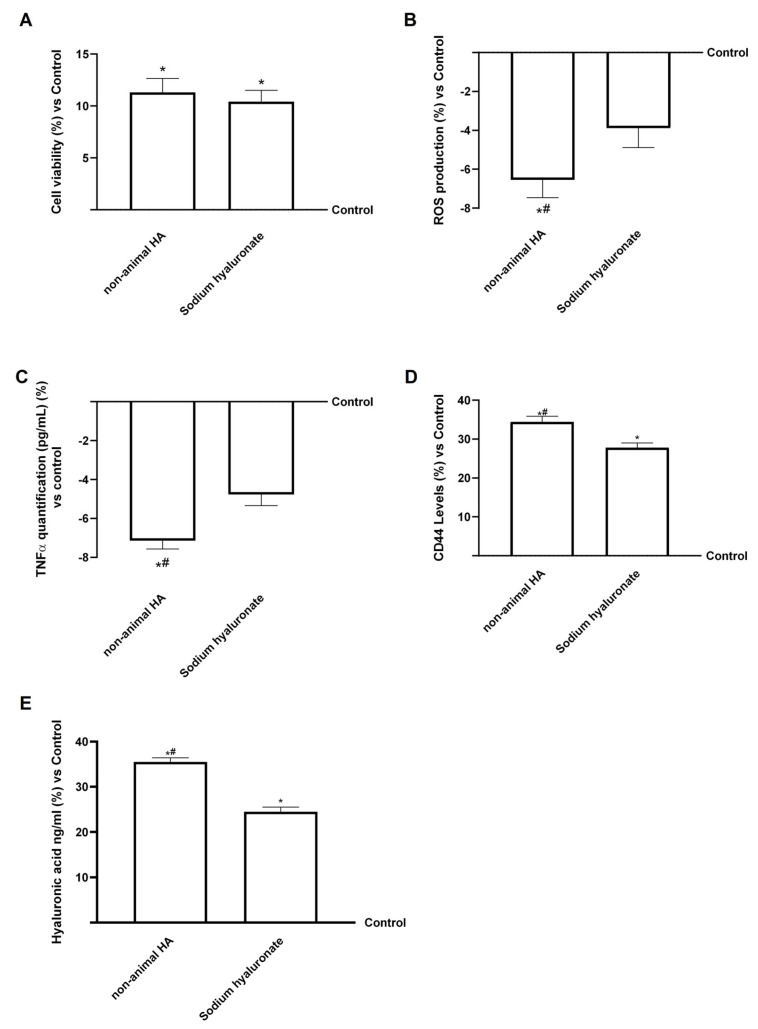
Biological analysis of fungal-derived HA and sodium hyaluronate at the keratinocyte level. (**A**) Cell viability assessed with MTT assay; (**B**) ROS production measured by cytochrome C reduction assay; (**C**) TNF-α levels determined via ELISA; (**D**) CD44 protein expression analysed by Western blot; (**E**) Intracellular and extracellular HA levels evaluated by ELISA. Data are presented as mean ± SD from five independent experiments conducted in triplicate, relative to control (0%). * *p* < 0.05 vs. control; # *p* < 0.05 vs. sodium hyaluronate.

To further explore the potential systemic biological effects of hepatic eluate fungal-derived HA, additional analyses were performed using an in vitro model of human keratinocytes. This model was employed to assess cellular viability, oxidative and inflammatory responses, CD44 receptor expression, and HA internalisation, thereby providing insight into the compound’s potential to support epidermal homeostasis and regeneration. As shown in Figure 12A, both hepatic eluate fungal-derived HA and sodium hyaluronate significantly increased keratinocyte viability compared with the control (*p* < 0.05). Fungal-derived HA showed a slightly greater effect, confirming its good cytocompatibility and potential to promote skin cell renewal. The evaluation of ROS production (Figure 12B) demonstrated that both compounds effectively reduced oxidative stress compared with the control (*p* < 0.05), even in this compartment. As observed before, hepatic eluate fungal-derived HA induced a more pronounced decrease (about 67% vs. sodium hyaluronate, *p* < 0.05), suggesting a stronger antioxidant potential and the ability to preserve redox balance within the epidermal environment. Similarly, TNFα quantification (Figure 12C) showed that both hepatic eluate fungal-derived HA and sodium hyaluronate significantly reduced pro-inflammatory cytokine production relative to the control (*p* < 0.05). Fungal-derived HA exhibited the most effective anti-inflammatory action (about 48% vs. sodium hyaluronate, *p* < 0.05), supporting its protective role against inflammation-mediated skin damage in vitro.

As illustrated in Figure 12D, both treatments significantly increased CD44 levels compared with the control (*p* < 0.05), with hepatic eluate fungal-derived HA showing greater upregulation than sodium hyaluronate (about 24%, *p* < 0.05). Since CD44 is the main receptor mediating HA internalisation, its upregulation indicates active HA–cell interaction. Consistently, the quantification of intracellular HA (Figure 12E) confirmed that hepatic eluate fungal-derived HA led to a greater accumulation of HA within keratinocytes compared with both the control and sodium hyaluronate (about 45% vs. sodium hyaluronate, *p* < 0.05). This enhanced HA internalisation represents a beneficial outcome, as it could support intracellular hydration mechanisms and contribute to epidermal renewal and repair.

In order to provide additional evidence for the greater activity of fungal-derived HA relative to sodium hyaluronate, the principal biological endpoints (cell viability, ROS production, and CD44 expression) were normalised to the effective content of pure HA. The adjusted outcomes demonstrated consistency (see Table A7 in Appendix A), indicating that the observed differences cannot be attributed solely to variations in purity.

**Figure 13:**
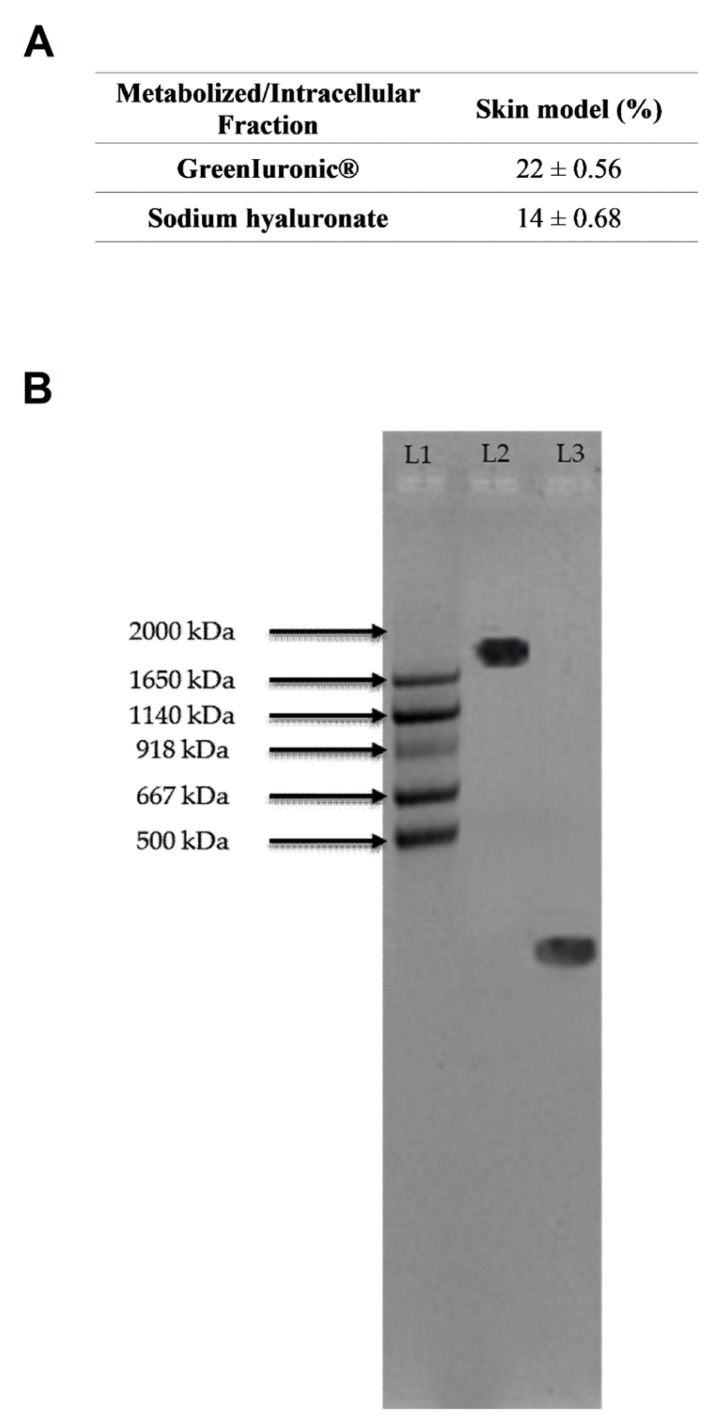
Quantification of HA content and molecular weight in the keratinocyte cells (HEKa). In (**A**), Total HA quantification (% *w*/*w*) based on glucuronic acid calibration curves. Data represent mean ± SD from five independent experiments performed in triplicate. In (**B**), Representative 1% agarose gel showing HA molecular weight distribution. MW: HA molecular weight standard; L1: ladder; L2: fungal-derived HA; L3: sodium hyaluronate.

Similarly to the previous procedure, the titre and molecular weight of both forms of hepatic eluate were also measured at the skin in vitro model to ensure that the amount reaching the target sites was well maintained and not degraded. As shown in Figure 13A and in Appendix A (Table A1), the data indicate that the intracellular fraction of the in vitro skin model contained HA at a titre of 22%, compared with 59% following hepatic metabolism. Additionally, only a negligible amount of sodium hyaluronate reaches the skin cells, with a titre of 14% compared to 35.9% after crossing hepatocytes.

In addition, molecular weight analysis (Figure 13B) confirmed that hepatic eluate fungal-derived HA maintains its high molecular weight HA (>2000 kDa), while hepatic eluate sodium hyaluronate maintains a molecular weight of <500 kDa.

The results of this study indicate that fungal-derived HA is present in the skin in vitro model in a structurally intact and bioactive form, thereby confirming its greater stability compared to sodium hyaluronate. The presence of high-molecular-weight HA in skin cells also suggests a potential role in supporting hydration and tissue regeneration, thereby highlighting the effectiveness of fungal-derived HA in maintaining its activity across several in vitro barrier models.

## 4. Discussion

HA has long been recognised as a key extracellular matrix component involved in tissue hydration, elasticity, and repair. It continues to attract growing scientific attention as both a therapeutic biopolymer and a nutraceutical ingredient [1]. Recent research has increasingly examined the systemic effects of orally administered HA; however, its metabolic fate, absorption mechanisms, and multi-organ biological activity remain inadequately characterised, particularly for emerging non-animal-derived HA variants. The present study provides a comprehensive evaluation of the metabolic fate and biological activity of a fungal-derived HA extracted from *Tremella Fuciformis*, using a sequential in vitro multi-barrier model that mimics the physiological passage from gastric to systemic distribution. This approach allowed us to examine, for the first time, the stability, absorption, hepatic handling, and biological effects of fungal-derived HA across different cellular environments representative of gastrointestinal, hepatic, cartilage, skin, and renal tissues. Although differences in purity may partially affect the observed biological responses, the structural properties of fungus-derived hyaluronic acid, including molecular weight and polymer integrity, appear to play a key role. Using sodium hyaluronate as a reference standard allowed for a comparative evaluation of the fungal-derived preparation under identical nominal conditions. In addition, the study was designed as a mechanistic investigation using a biologically validated concentration rather than a dose–response or pharmacokinetic study. The initial challenge for HA is maintaining molecular integrity in the gastric and intestinal environments. In the gastric in vitro model, fungal-derived HA preserved cell viability and epithelial integrity while limiting oxidative and inflammatory stress, indicating strong acid stability. Additional analysis shows that fungal-derived HA maintains a high titre of approximately 85%, with a reduction of approximately 5% compared to the amount administered.

In contrast, sodium hyaluronate shows a reduction of approximately 6.3%. This smaller reduction in titre could suggest that fungal-derived HA has greater intrinsic resistance to the gastric environment than sodium hyaluronate. The molecular weight assessment also revealed the structural resistance of fungal-derived HA after crossing the gastric in vitro barrier. Moreover, fungal-derived HA was less retained intracellularly and more efficiently transferred across the gastric barrier, which may facilitate its subsequent intestinal absorption in vitro.

In the intestinal model, fungal-derived HA more effectively preserved barrier integrity and reduced inflammation, supporting previous evidence linking HA to the modulation of epithelial junctions and inflammatory signalling pathways involved in mucosal protection [55]. The data obtained in this environment indicate that fungal-derived HA not only remains stable but may also contribute to epithelial protection. Further, the lower induction of CD44 expression observed for fungal-derived HA compared with sodium hyaluronate may reflect a more efficient translocation process rather than receptor-mediated uptake. These observations align with previous evidence showing that orally administered HA resists gastrointestinal degradation, undergoes colonic absorption, and subsequently distributes to peripheral tissues [6].

Regarding the titre and molecular weight of the two forms of HA, an approximate reduction of 11% for fungal-derived HA and 9.5% for sodium hyaluronate was observed between the gastric and intestinal compartments. This decline is presumably attributable to degradation occurring during transit through the intestinal environment. Nevertheless, the limited, nearly identical reduction observed in both formulations suggests that they can cross the gastric phase while maintaining adequate structural stability. Notably, the residual content of fungal-derived HA has been shown to exhibit enhanced resistance to acid degradation and to preserve high molecular weight. These characteristics may facilitate more efficient intestinal absorption and increased systemic availability in subsequent stages of metabolism in vitro.

The data obtained from the gastric to hepatic compartments are consistent with those reported in the literature on oral administration, thereby confirming the validity of the models used and the analysis protocol employed [56]. However, the hepatic model analysis showed reduced intracellular uptake and greater extracellular persistence of fungal-derived HA compared with sodium hyaluronate, suggesting that a larger portion may transit to peripheral tissues. This finding is consistent with preclinical evidence showing high-MW HA was detected in connective tissues 24 h after oral administration in animal models [57]. In this district, the titre and molecular weight of the two forms of HA are reduced by approximately 15% for fungal-derived HA and 13% for sodium hyaluronate in the transition from the intestinal to the hepatic environment. This reduction can be attributed to normal liver metabolic processes, during which part of the compound is used or transformed. However, the stability of the molecular weight observed for both forms indicates that the hyaluronic acid structure does not undergo significant alterations, suggesting that hepatic metabolism mainly affects the available quantity rather than the integrity of the molecule.

After characterising the hepatic compartment, further experiments focused on the renal compartment to assess excretion mechanisms [58]. Both fungal-derived HA and sodium hyaluronate preserved cell viability and reduced oxidative and inflammatory stress, confirming their safety at the renal level. However, while sodium hyaluronate led to higher excreted HA levels, fungal-derived HA showed reduced extracellular HA levels, indicating more efficient systemic retention. These results are consistent with the hypothesis that fungal-derived HA could persist longer in the medium, thereby prolonging biological effects. Both HA forms exhibited lower titres than those observed in the hepatic compartment, decreasing by approximately 52% for fungal-derived HA and around 33% for sodium hyaluronate. This marked decrease suggests that, following hepatic metabolism, fungal-derived HA was predominantly distributed. This hypothesis is further substantiated by molecular weight analysis, which revealed that the low HA concentration in the renal cells precluded accurate determination of its MW. Because HA levels detected in the renal compartment were extremely low, reliable molecular weight analysis was not feasible without risking structural artefacts. This finding suggests two potential mechanisms: firstly, partial degradation of hyaluronic acid during excretion; secondly, enhanced retention of the high-molecular-weight fraction of fungal-derived HA in target tissues. After hepatic metabolism and excretion, the resulting compounds can be investigated at peripheral targets, such as skin and joint tissues, in other in vitro models.

HA is essential for maintaining the integrity of connective tissues, particularly in joints; indeed, numerous studies demonstrate HA’s potential for treating joint disorders. The main mechanism of HA’s effectiveness is chondroprotection, supported by other mechanisms, such as anti-inflammatory effects [59]. The analysis of TNFα production at the chondrocyte level showed that fungal-derived HA retained anti-inflammatory activity after gastrointestinal and hepatic passage, indicating the positive effects of this fungal-derived HA. Further, one of the main molecular mechanisms underlying HA chondroprotective action is its binding to CD44 [59]. It strongly increased CD44 levels in chondrocytes, demonstrating that it is correctly internalised into the cells, where it can exert its beneficial effects. It is worth noting that fungal-derived HA maintained its high MW, data of particular importance, as research comparing HA of different MWs showed that, when it comes to treating joint disorders, higher MW HA had more positive effects and outcomes than lower MW HA [59]. In this district, our results suggested that fungal-derived HA could modulate inflammation even after absorption, metabolism, and excretion, reducing TNFα production, as reported in the literature [35]. Fungal-derived HA interacted with CD44 on chondrocytes after gastrointestinal transit and hepatic processing, consistent with HA–CD44–mediated mechanisms associated with improved absorption and decreased proinflammatory signalling [60]. Another aspect that emerged from this study is the behaviour of hyaluronidase enzymes in simulated physiological compartments. Hyaluronidases are key regulators of HA turnover in tissues such as the liver and kidney, where they mediate its degradation into low-molecular-weight fragments. Their upregulation is often linked to stress or tissue damage [8]. In the multibarrier model, hyaluronidase activity remained stable across intestinal, hepatic, and renal compartments. It was not increased by either HA form, indicating that endogenous HA-degrading pathways were not activated. Fungal-derived HA has been shown to cause a modest decrease in enzymatic activity in hepatic and renal cells, consistent with its enhanced extracellular stability and reduced intracellular uptake. The unchanged hyaluronidase profile suggests that fungal-derived HA is less susceptible to enzymatic hydrolysis, thereby supporting its greater persistence along the simulated metabolization and excretion pathway and its potential for enhanced systemic activity.

In addition, some studies have demonstrated that daily HA supplement consumption can positively affect skin cells, improving cell homeostasis [61,62]. HA-based formulations demonstrate anti-inflammatory efficacy in inflammatory skin conditions [63]. Consistently, fungal-derived HA reduced TNFα production more effectively than sodium hyaluronate in the present study. HA has also been associated with enhanced keratinocyte proliferation and increased epidermal thickness [64]. Additionally, the interaction between HA and CD44 provides effective methods for treating a range of skin conditions associated with ageing [65]. In the current study, fungal-derived HA strongly increased CD44 levels in keratinocytes, demonstrating that it is correctly internalised into the cells while maintaining its high MW, an important factor, as high MW HA has already been demonstrated to promote keratinocyte health with a more positive effect [12].

In conclusion, the analysis of HA titre and molecular weight in chondrocytes and keratinocytes confirmed the greater stability and bioavailability of fungal-derived HA compared with sodium hyaluronate. In both cell types, fungal-derived HA maintained a higher molecular weight and titre, suggesting reduced degradation and more efficient distribution to target tissues. Furthermore, analysis of HAS isoforms demonstrated activation of endogenous HA synthesis in chondrocytes, as evidenced by a marked upregulation of HAS2, while HAS3 expression remained unchanged. This pattern is consistent with the literature identifying HAS2 as the predominant isoform responsible for high-molecular-weight HA production in cartilage, indicating stimulation of biologically functional HA synthesis rather than passive extracellular accumulation [66]. The absence of HAS3 induction further supports the interpretation of a homeostatic response, rather than one associated with inflammatory signalling or rapid tissue remodelling, following fungal-derived HA treatment [67,68].

Despite the apparent ambiguity in CD44 expression in different tissues, this evidence is consistent with the extant literature. In clearance organs, such as the liver, the HA–CD44 interaction is primarily associated with internalization and metabolic turnover [69]. Therefore, reduced expression may favour systemic persistence. In peripheral tissues, such as cartilage and skin, CD44 plays a functional signalling role: in chondrocytes, it regulates the extracellular matrix and modulates inflammation, and in keratinocytes, it stimulates proliferation and tissue repair [70,71]. The increased expression and internalisation of CD44 in these tissues therefore indicate greater functional bioactivity, consistent with the different biology of CD44 in the various compartments.

Overall, these findings suggest that fungal-derived HA may improve persistence and biological function in both articular and cutaneous compartments. Taken together, these findings demonstrate a consistent pattern in which fungal-derived HA exhibits improved stability, antioxidant and anti-inflammatory properties. They may suggest greater systemic availability under in vitro conditions than sodium hyaluronate. The ability of fungal-derived HA to preserve its molecular integrity in the gastric and hepatic environments, efficiently cross the intestinal barrier, and reach target tissues. This study, for the first time, provides evidence of the systemic metabolism of fungal-derived HA using a sequential in vitro multi-barrier model that reproduces the physiological progression of absorption, as revealed by greater data homology with the literature. This approach allowed the assessment of compound stability, transfer dynamics, and biological effects within a systemic-like context. In addition, when data were normalised to the effective amount of pure HA, the overall trends remained consistent, supporting the interpretation that qualitative molecular features contribute to the observed biological profile.

While the in vitro models employed permitted the reconstruction of the passage of HA formulations through different tissue compartments, it is important to note that these results offer only preliminary mechanistic evidence. Confirmation of extended circulation time and systemic availability will necessitate dedicated in vivo pharmacokinetic studies. Despite the validation of the Caco-2 model in the study of intestinal HA absorption, the exclusion of colonic fermentation from the current model may be considered as a potential area for future study, with a view to the full simulation of the metabolic fate of HA. The present work should therefore be viewed as a mechanistic ADME-focused in vitro investigation rather than a pharmacokinetic or clinical efficacy study.

Nevertheless, certain limitations should be acknowledged, including the inability to fully capture the complexity of in vivo processes such as enzymatic degradation by microbiota, hormonal regulation, and tissue-specific uptake kinetics. Future integration of this model may better simulate dynamic fluidic conditions and inter-organ communication. Further in vivo investigations are warranted to confirm the systemic distribution and pharmacodynamic effects suggested by the present in vitro results.

## 5. Conclusions

The present study demonstrates that fungal-derived HA, a plant-derived hyaluronic acid obtained from White Tremella, exhibits improved transepithelial transfer in vitro and antioxidant and anti-inflammatory effects across multiple biological barriers in vitro. In comparison with sodium hyaluronate, fungal-derived HA demonstrated superior systemic transfer, greater extracellular availability, and increased activity on target cells, including chondrocytes and keratinocytes. The results of this study suggest that it maintains biological activity in vitro, as demonstrated by increased CD44 receptor expression and HA internalisation.

These findings provide valuable mechanistic insight into the systemic behaviour of fungal-derived HA under in vitro conditions. However, it should be noted that the observed enhanced systemic transfer and persistence are derived from sequential in vitro models; therefore, direct pharmacokinetic validation in vivo is necessary to confirm these effects.

These findings suggest that fungal-derived HA is a sustainable and promising alternative for nutraceutical and therapeutic strategies targeting joint and skin health, although in vivo and clinical studies are required to establish dosing, bioavailability, and clinical efficacy.

## Data Availability

Data are available from the corresponding author upon reasonable request and for justified scientific reasons, since they relate to a patented substance.

## Abbreviations

The following abbreviations are used in this manuscript:

Adv DMEM	Advanced Dulbecco’s Modified Eagle Medium
Adv DMEM/F12	Advanced Dulbecco’s Modified Eagle’s Medium/Nutrient F-12 Ham
ATCC	American Type Culture Collection
CD44	Cluster of differentiation 44
DMEM	Dulbecco’s Modified Eagle’s Medium
ECM	Extracellular matrix
FBS	Fetal bovine serum
HA	Hyaluronic acid
HAase	Hyaluronidase
HCl	Hydrochloric acid
HYAL1	Hyaluronidase 1
HYAL2	Hyaluronidase 2
MW	Molecular weight
PBS	Phosphate-Buffered Saline
PET	Translucent polyethene terephthalate
PVDF	Polyvinylidene fluoride
RIPA	Radioimmunoprecipitation assay
ROS	Radical oxygen species
SD	Standard deviation
SDS-PAGE	Sodium Dodecyl Sulphate-PolyAcrylamide Gel Electrophoresis
TAE	Tris-acetate-EDTA
TEER	Transepithelial electrical resistance
TFP	Tremella fuciformis
TNFα	Tumour Necrosis Factor α

## Appendix A

**Table A1:** HA Titer of fungal-derived HA and Sodium Hyaluronate in In Vitro Compartment Models. The data set includes the percentage titer of hyaluronic acid (HA) in fungal-derived HA and sodium hyaluronate samples after exposure to various biological models, including the stomach, intestines, liver, kidneys, joints, and skin. The results are expressed as means (%) ± SD of five independent experiments performed in triplicate.

Sample	Administration (%)	Gastric Model (%)	Intestinal Model (%)	Hepatic Model (%)	Renal Model (%)	Joint Model (%)	Skin Model (%)
Fungal-derived HA	90.5 *±* 1.49	85 *±* 1.03	74 *±* 0.98	59 *±* 0.82	7 *±* 0.15	64 *±* 1.27	22 *±* 0.56
Sodium hyaluronate	65 *±* 1.54	58.7 *±* 1.12	49.2 *±* 1.02	35.9 *±* 0.94	2.5 *±* 0.17	40 *±* 1.30	14 *±* 0.68

**Table A2:** Results of key biological endpoints (cell viability, ROS production, and CD44 expression) normalised to the actual amount of pure HA in the gastric compartment.

MTT	1 h	2 h	3 h	4 h
Fungal-derived HA	10.8 ± 0.64	14.9 ± 0.97	24.9 ± 1.02	30.8 ± 1.20
Sodium hyaluronate	7.5 ± 0.43	13.9 ± 0.90	18.9 ± 1.01	31.5 ± 1.35
**ROS**	**1 h**	**2 h**	**3 h**	**4 h**
Fungal-derived HA	−1.9 ± 0.03	1.4 ± 0.03	2.7 ± 0.03	4.3 ± 0.03
Sodium hyaluronate	−1.6 ± 0.03	3.6 ± 0.03	3.9 ± 0.03	6.3 ± 0.03
**CD44**	**4 h**			
Fungal-derived HA	2.0 ± 0.03			
Sodium hyaluronate	6.5 ± 0.12			

**Table A3:** Results of key biological endpoints (cell viability, ROS production, and CD44 expression) normalised to the actual amount of pure HA in the intestinal compartment.

MTT	2 h	3 h	4 h	5 h	6 h
Fungal-derived HA	5.1 ± 0.13	8.2 ± 0.24	16.9 ± 1.12	12.7 ± 1.07	5.1 ± 0.16
Sodium hyaluronate	5.4 ± 0.15	7.1 ± 0.21	13.4 ± 1.05	11.6 ± 0.98	6.7 ± 0.19
**ROS**	**2 h**	**3 h**	**4 h**	**5 h**	**6 h**
Fungal-derived HA	−16.0 ± 1.51	−12.9 ± 1.27	−8.5 ± 0.92	−5.9 ± 0.73	−3.9 ± 0.16
Sodium hyaluronate	−11.3 ± 1.34	−8.5 ± 0.89	−3.2 ± 0.10	−0.3 ± 0.01	4.2 ± 0.26
**CD44**	**6 h**				
Fungal-derived HA	7.2 ± 0.44				
Sodium hyaluronate	11.5 ± 1.09				

**Table A4:** Results of key biological endpoints (cell viability, ROS production, and CD44 expression) normalised to the actual amount of pure HA in the hepatic compartment.

Sample	MTT	ROS	CD44
Fungal-derived HA	7.1 ± 0.33	−9.1 ± 0.82	11.9 ± 1.19
Sodium hyaluronate	6.5 ± 0.28	−5.3 ± 0.61	19.5 ± 1.75

**Table A5:** Results of key biological endpoints (cell viability, ROS production, and CD44 expression) normalised to the actual amount of pure HA in the renal compartment.

Sample	MTT	ROS	CD44
Fungal-derived HA	6.1 ± 0.50	−7.9 ± 0.66	2.6 ± 0.12
Sodium hyaluronate	5.6 ± 0.47	−4.4 ± 0.32	4.9 ± 0.26

**Table A6:** Results of key biological endpoints (cell viability, ROS production, and CD44 expression) normalised to the actual amount of pure HA in chondrocytes.

Sample	MTT	ROS	CD44
Fungal-derived HA	11.0 ± 0.94	−7.7 ± 0.31	55.7 ± 2.05
Sodium hyaluronate	6.7 ± 0.52	−3.4 ± 0.23	44.2 ± 1.96

**Table A7:** Results of key biological endpoints (cell viability, ROS production, and CD44 expression) normalised to the actual amount of pure HA in keratinocytes.

Sample	MTT	ROS	CD44
Fungal-derived HA	10.9 ± 0.37	−6.9 ± 0.28	34.0 ± 1.83
Sodium hyaluronate	10.4 ± 0.34	−3.9 ± 0.15	27.8 ± 1.72
